# Conditional Wave Function Theory: A Unified Treatment of Molecular Structure and Nonadiabatic Dynamics

**DOI:** 10.1021/acs.jctc.1c00772

**Published:** 2021-11-09

**Authors:** Guillermo Albareda, Kevin Lively, Shunsuke A. Sato, Aaron Kelly, Angel Rubio

**Affiliations:** † Nano-Bio Spectroscopy Group and European Theoretical Spectroscopy Facility (ETSF), Universidad del País Vasco (UPV/EHU), Av. Tolosa 72, 20018 San Sebastian, Spain; ‡ Institute of Theoretical and Computational Chemistry, University of Barcelona, Martí i Franquès 1-11, 08028 Barcelona, Spain; § Max Planck Institute for the Structure and Dynamics of Matter and Center for Free-Electron Laser Science, Luruper Chaussee 149, 22761 Hamburg, Germany; ∥ The Hamburg Centre for Ultrafast Imaging, University of Hamburg, Luruper Chaussee 149, 22761 Hamburg, Germany; ⊥ Center for Computational Sciences, University of Tsukuba, 1-1-1 Tennodai, Tsukuba, Ibaraki 305-8577, Japan; # Department of Chemistry, Dalhousie University, Halifax, Nova Scotia B3H 4R2, Canada; ∇ Center for Computational Quantum Physics (CCQ), Flatiron Institute, 162 Fifth Avenue, New York, New York 10010, United States

## Abstract

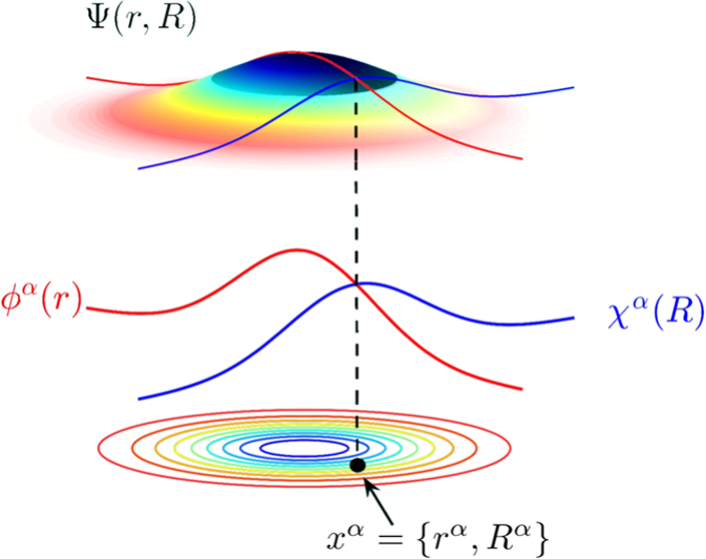

We demonstrate that a conditional wave function theory enables a unified and efficient treatment of the equilibrium structure and nonadiabatic dynamics of correlated electron–ion systems. The conditional decomposition of the many-body wave function formally recasts the full interacting wave function of a closed system as a set of lower-dimensional (conditional) coupled “slices”. We formulate a variational wave function ansatz based on a set of conditional wave function slices and demonstrate its accuracy by determining the structural and time-dependent response properties of the hydrogen molecule. We then extend this approach to include time-dependent conditional wave functions and address paradigmatic nonequilibrium processes including strong-field molecular ionization, laser-driven proton transfer, and nuclear quantum effects induced by a conical intersection. This work paves the road for the application of conditional wave function theory in equilibrium and out-of-equilibrium ab initio molecular simulations of finite and extended systems.

## 1 Introduction

Emerging experimental capabilities in the precise manipulation of light and matter are opening up new possibilities to understand and exploit correlations and quantum effects that can be decisive in the functional properties of molecules and materials. Light-driven states can not only be designed to monitor and/or control the structure of molecules^1−7^ and solids^8−12^ but also form light–matter hybrid states with new physical properties.^13−21^ In view of these exciting developments, accurate first-principles theoretical techniques are also needed to help interpret observations, to enable the predictions of simplified models to be scrutinized, and, ultimately, to help gain predictive control. Our ability to treat the full correlated quantum structure and dynamics of general electron–ion systems unfortunately remains limited by the unfavorable scaling of the many-body problem.

A standard approach to address this problem in molecular and solid-state systems has been to “divide-and-conquer” in the sense that the electronic structure and the electron–nuclear interactions are treated separately. Introduced almost a century ago by Born and Oppenheimer,^22^ the adiabatic approximation, i.e., the assumption that electrons adjust instantaneously to the motion of nuclei, is the cornerstone of this so-called standard approach. The Born–Oppenheimer (BO) approximation has been crucial to the development of a vast majority of approaches in quantum chemistry and condensed-matter theory,^23,24^ and the concept of ground-state Born–Oppenheimer potential-energy surface (BOPES) is the foundation for understanding the properties of systems at thermal equilibrium such as chemical reactivity^25−27^ and nuclear quantum effects,^28−31^ as well as of systems driven out of equilibrium.^32−35^

Accurately describing systems driven away from equilibrium and including nonadiabatic electron–nuclear effects places even more stringent demands on the development of practical first-principles tools. In the standard approach, one directly builds upon the BO approximation by expanding the full molecular wave function in the Born–Huang basis.^36^ Within this framework, nonadiabatic processes can be viewed as nuclear wavepacket dynamics with contributions on several BOPESs, connected through nonadiabatic coupling terms that induce electronic transitions.^37^ In this picture, trajectory-based quantum dynamics methods offer a trade-off between physical accuracy and computational cost.^38−40^ Of these approaches, perhaps the most popular are the Ehrenfest mean-field theory^41^ and Tully’s surface hopping dynamics.^42^ Both of these approaches consist of an ensemble of uncorrelated trajectories. Reintroducing correlation, for example, using a variety of wave function ansatz,^43−48^ semiclassical techniques,^49,50^ the quantum-classical Liouville equation,^51−53^ path-integral methods,^54,55^ or methods based on the exact factorization,^56−58^ allows for further accuracy with increased computational effort.

While advances in the ab initio electronic structure theory in quantum chemistry and condensed matter have made computing the ground-state energies both routinely efficient and rather accurate in many cases, obtaining accurate excited-state information remains a challenging problem in its own right. Even in cases where the excited-state electronic structure is available, performing fully quantum nuclear dynamics calculations using the standard approach quickly becomes infeasible^35,43^ as the memory required to store the information contained in the BOPESs grows rapidly with the number of correlated degrees of freedom. In this respect, gaining the ability to rigorously treat selected nuclear degrees of freedom quantum mechanically without incurring an overwhelming computational cost is the goal.

An alternative approach for describing quantum effects in coupled electron–ion systems is using a real-space representation of all degrees of freedom. This route might sound less intuitive as it avoids routine concepts such as BOPESs and nonadiabatic couplings that are fundamental in the present description and understanding of quantum molecular dynamics. However, this feature might be turned into an attractive playground from the computational point of view, as these quantities are usually demanding to obtain and fit from ab initio electronic structure calculations. In this framework, one of the leading approximate methods to describe the coupled electron–nuclear dynamics for large systems is time-dependent density functional theory coupled to classical nuclear trajectories through the Ehrenfest method.^60^ Due to its favorable system-size scaling, the real-space picture Ehrenfest method has been successful for a great many applications, from capturing phenomena associated with vibronic coupling in complex molecular systems^61^ and photodissociation dynamics in small molecules^62^ to radiation damage in metals;^63^ its efficiency allows calculations on large systems for even hundreds of femtoseconds.^64^ It has also been recently combined with the nuclear-electronic orbital method as a way to include quantum effects for selected nuclear degrees of freedom to study proton transfer processes in molecular excited states.^65^

It is well known, however, that the Ehrenfest approach can be inaccurate due to its mean-field nature. One classic example of this breakdown occurs in photochemical reaction dynamics, where mean-field theory can often fail to correctly describe the product branching ratios.^39,66^ Generally speaking, the mean-field description of any transport property can potentially suffer some deficiency; this is sometimes referred to as a violation of detailed balance,^67^ but it ultimately stems from the lack of time-translational invariance that is inherent to any approximate method that does not rigorously preserve the quantum Boltzmann distribution.^68^

The conditional wave function (CWF) framework introduced in ref (69) offers a route to go beyond the limits of mean-field theory while retaining a real-space picture; it is an exact decomposition and recasting of the total wave function of a closed quantum system.^70^ When applied to the time-dependent Schrödinger equation, the conditional decomposition yields a set of coupled, non-Hermitian, equations of motion.^69^ One can draw connections between CWF theory and other formally exact frameworks proposed in the literature to develop novel approximate schemes that provide a completely new perspective to deal with the long-standing problems of nonadiabatic dynamics of complex interacting systems.^71,72^ An example is the time-dependent interacting conditional wave function approach (ICWF),^73,74^ a recently introduced method for performing quantum dynamics simulations that is multiconfigurational by construction. Using a stochastic wave function ansatz that is based on a set of interacting single-particle CWFs, the ICWF method is a parallelizable technique, which achieves quantitative accuracy for situations in which mean-field theory drastically fails to capture qualitative aspects of the dynamics, such as quantum decoherence, using orders of magnitude fewer trajectories than the converged mean-field results.^73^

In this work, we introduce an exact time-independent version of the CWF mathematical framework. The time-independent CWF framework is formulated in real space, and it is an exact decomposition of the time-independent wave function of a closed quantum system that yields a set of coupled nonlinear eigenvalue problems and associated conditional eigenstates. Based on this framework, we put forth a static-basis version of the ICWF method, which allows us to establish an efficient and accurate algorithm for calculating the ground- and excited-state structures of correlated electron–nuclear systems and eventually extended systems. Importantly, the combination of the static version of the ICWF method using a time-dependent conditional eigenstate basis sets the stage for the implementation of a general-purpose ab initio molecular simulator that is formulated in the real-space picture and that self-consistently treats stationary states, as well as driven dynamics.

This manuscript has the following structure: in Section 2, we define the mathematical structure of the time-independent version of the CWF framework. Based on these results, we put forth an imaginary-time version of the ICWF technique in Section 3 for solving the time-independent Schrödinger equation and the performance of the resulting algorithm is assessed through the calculation of the ground-state and the low-lying excited-state BOPESs of the hydrogen molecule in one dimension (1D). In Section 4, a real-time extension of this multiconfigurational ansatz is presented, along with an algorithm for solving the time-dependent Schrödinger equation using a stochastic static-basis ansatz. The ability of the resulting algorithm in capturing static and dynamic properties is then assessed by evaluating the absorption spectrum and a laser-induced dynamics of the aforementioned H_2_ model system. In Section 5, we revisit the exact time-dependent CWF framework, and in Section 6, we present the dynamical ICWF (dyn-ICWF) approach to the time-dependent Schrödinger equation. The performance of the time-dependent ICWF method in combination with its imaginary-time variation for preparing the initial state is demonstrated for three model systems, viz., a laser-driven proton-coupled electron transfer model, an electron-atom scattering process, and an example of nuclear quantum effects in the dynamics through a conical intersection (CI). A summary of the main results of this work and an outlook on future directions are offered in Section 7.

## 2 Conditional Eigenstates

We begin by considering a closed system with *n* electrons and *N* nuclei, collectively denoted by ***x*** = (***r***, ***R***). We use the position representation for both subsets; lowercase symbols will be used for the electronic subsystem, e.g., ***r*** = {***r***_1_*s*_1_, ..., ***r***_*n*_*s*_*n*_}, and uppercase symbols ***R*** = {***R***_1_σ_1_, ..., ***R***_*N*_σ_*N*_} for the nuclear subsystem. Hereafter, electronic and nuclear spin indices, respectively, *s*_*j*_ and σ_*j*_, will be made implicit for notational simplicity, and, unless otherwise stated, all expressions will be given in atomic units.

The time-independent CWF can be constructed starting from the nonrelativistic time-independent Schrödinger equation in the position representation
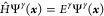
1where Ψ^γ^(***x***) is an eigenstate of the molecular Hamiltonian *Ĥ* with label γ and the corresponding energy eigenvalue *E*^γ^. The molecular Hamiltonian operator *Ĥ* in eq 1 can be written as
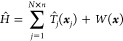
2where the kinetic energy operators are , *m*_*j*_ and *z*_*j*_ being the characteristic mass and charge of particle *j*, respectively. The full electron–nuclear potential energy of the system is *W*(***x***) (written in the position basis rather than, say, the BO or Born–Huang basis), and **A** is the vector potential due to an arbitrary static external electromagnetic field.

Note that the total Hamiltonian in eq 1 is invariant under translations and rotations of all particles. This means that the eigenstates of the system will be invariant under transformations by the translation and rotation groups. Together with the inversion symmetry, this implies that all one-body quantities such as the electron density or any nuclear-reduced density are constant and that two-particle position correlation functions only depend on the distance between their arguments. This is obviously not a convenient starting point to describe the structure of a quantum system. The solution to this problem relies on transforming the Hamiltonian to a fixed coordinate system that reflects the internal properties of the system.^a^ This is, in general, not a trivial task, and hereafter, we will assume that eq 1 already reflects such internal properties, either by exploiting a particular symmetry of the system or by simply introducing a parametric dependence on, e.g., a fixed (heavy) nuclear position.

**Figure 1:**
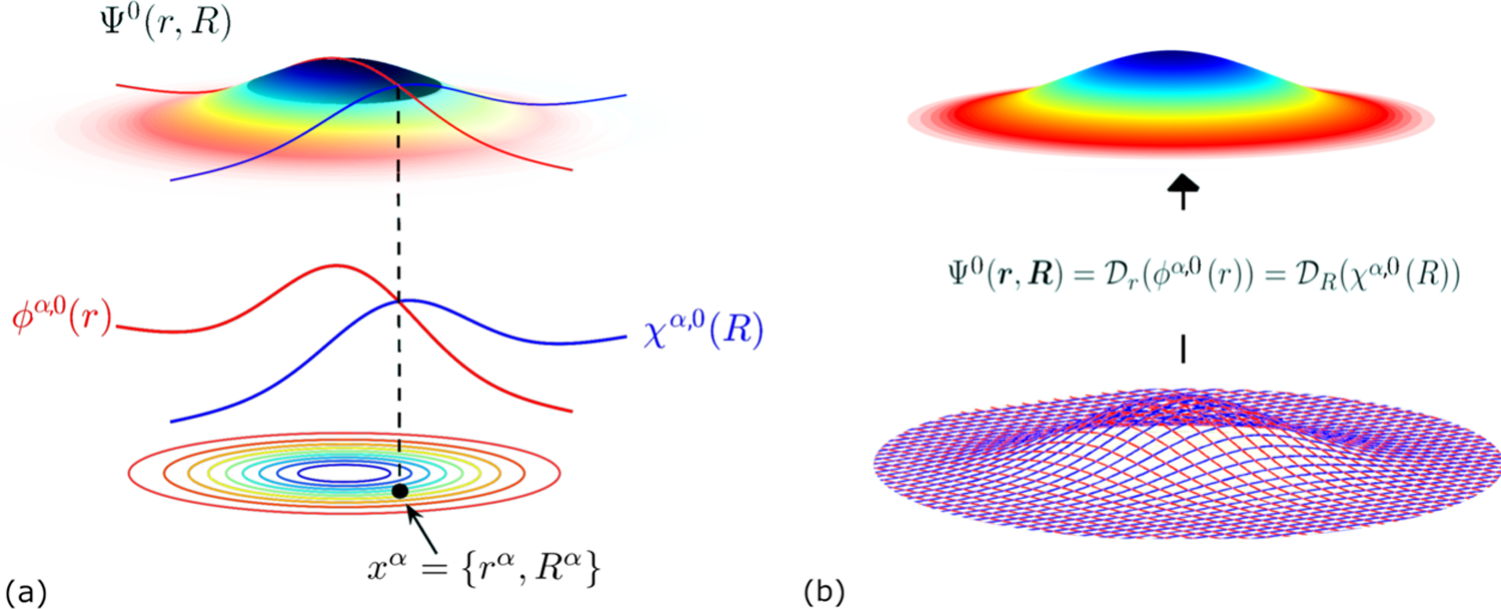
Schematic representation of the CWF approach to the time-independent Schrödinger equation for one electron and one nucleus in one dimension, i.e., ***x*** = (*r*, *R*). (a) The full ground-state Ψ^0^(*r*, *R*) is plotted together with a pair of conditional ground states ϕ^α,0^(*r*) for the electronic degree of freedom (in red) and χ^α,0^(*R*) for the nuclear degree of freedom (in blue) for a given position of the full configuration space {*r*^α^, *R*^α^}. Contour plots of the molecular wave function are also shown for clarity. (b) The exact solution of the time-independent Schrödinger equation in eq 1 can be reconstructed provided a sufficiently large ensemble of sampling points *x*^α^ = {*r*^α^, *R*^α^}. This can be done by applying the reassembling transformation  or  (whose definition can be found in Appendix A) to the ensemble of electronic ϕ^α,0^(*r*) or nuclear χ^α,0^(*R*) conditional eigenstates, respectively.

At this point, we can decompose the eigenstates Ψ^γ^(***x***) in terms of single-particle conditional eigenstates of either of the two subsystems, which are defined as follows
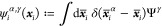
3Here, the index α denotes the particular conditional slice and ***x*®**_*i*_ = (***x***_1_, ..., ***x***_*i*–1_, ***x***_*i*+1_, ..., ***x***_*n*×*N*_) are the coordinates of all degrees of freedom in the system except ***x***_*i*_. Similarly, ***x*®**_*i*_^α^ = (***x***_1_^α^, ..., ***x***_*i*–1_^α^, ***x***_*i*+1_^α^, ..., ***x***_*n*×*N*_^α^) are some particular positions of all system degrees of freedom except ***x***_*i*_. As shown schematically in Figure 1, the conditional eigenstates in eq 3 represent one-body slices of the full many-body eigenstates Ψ^γ^(***x***) taken along the coordinate of the *i*th degree of freedom. The particle placement ***x***^α^ defining the CWFs has not yet been specified, and although, in principle, it can be chosen arbitrarily, it will be proven convenient in practice to exploit important sampling techniques.

Evaluating eq 1 at ***x*®**_*i*_^α^ by applying the integral operator in eq 3 yields conditional eigenstates that are the solutions of the following eigenvalue problem
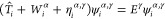
4where we introduced *W*_*i*_^α^(***x***_*i*_) = *W*(***x***_*i*_,***x*®**_*i*_^α^), with *W*(***x***) being the full electron–nuclear interaction potential appearing in the Hamiltonian of eq 2. In addition, η_*i*_^α,γ^(***x***_*i*_) are the kinetic correlation potentials given by
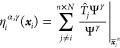
5

Provided a large enough collection of CWFs satisfying eq 4, an exact solution of eq 1 can be reconstructed by undoing the conditional decomposition of eq 3 (see Figure 1b).^69^ That is, given a set of conditional slices that sufficiently span the support of Ψ^γ^, then the corresponding conditional eigenstates can be used to reassemble the full electron–nuclear wave function
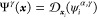
6using the transformations , which are discussed in more detail in Appendix A. This expression, eq 6, can be used to evaluate the kinetic correlation potentials in eq 5. In this way, the generalized one-body eigenvalue problem in eq 4 can be understood as an exact decomposition and recasting of the eigensolution of the full electron–nuclear system, which yields a set of coupled, non-Hermitian, eigenvalue problems.

### 2.1 Time-Independent Hermitian Approximation

An approximate solution to eq 4 can be formulated by expanding the kinetic correlation potentials around the sampling coordinates ***x***^α^ using Taylor series and then truncating at zeroth order, i.e.
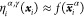
7At this level, the kinetic correlation potentials engender only a global phase that can be simply omitted as expectation values are invariant under such global phase transformations. Note that these approximated kinetic correlation potentials can be alternatively obtained by introducing a mean-field ansatz Ψ^γ^(***x***) = ∏_*i*=1_^*n*×*N*^ψ(***x***_*i*_) into eq 5. By making this approximation, the eigenvalue problems in eq 4 are restored to a Hermitian form
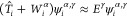
8The Hermitian limit allows the full many-body problem to be approximated as a set of independent single-particle problems. That is, the superscript γ refers exclusively to the conditional eigenstate excitation number.

## 3 Static Properties with Conditional Eigenstates

In general, the higher-order terms in the Taylor expansion of the kinetic correlation potentials are non-negligible. However, one can still take advantage of the simple Hermitian form of the conditional eigenvectors (hereafter referred to as conditional wave functions (CWFs)) in eq 8 to design an efficient many-body eigensolver by utilizing them as bases for electronic and nuclear degrees of freedom in a variational wave function ansatz.

While there is a diverse literature spanning decades on different forms for variational electron–nuclear wave function ansatz, for illustrative (and practical) purposes, we employ a sum-of-product form, which in the language of tensor decompositions is referred to as the canonical format.^77^ For each degree of freedom ***x***_*i*_, we utilize a given electronic or nuclear CWF, respectively, coming from solutions to eq 8, to approximate the γth full system exact excited state as follows
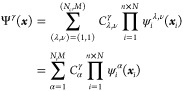
9where in the second line, we have rearranged the sum over particle position λ ∈ {1, ..., *N*_*c*_} and excited CWF ν ∈ {1, ..., *M*} into a single index α = λ + *N*_*c*_(ν – 1), such that α ∈ {1, ..., *N*_*c*_*M*}. The particle placement ***x***^α^ defining the conditional potentials *W*_*i*_^α^ has not yet been specified, and, in principle, it can be chosen arbitrarily; however, in practice, we choose to sample from initial guesses for the reduced densities of the electronic and nuclear subsystems.

We refer to this ansatz (eq 9) as being in canonical format because we do not mix all possible CWFs ψ_*i*_^λ,ν^ for all possible degrees of freedom ***x***_*i*_, as one does with a single-particle function bases across the different system degrees of freedom in the Tucker format employed in the multiconfigurational time-dependent Hartree (Fock)—MCTDH(F)^43^ and multiconfigurational electron–nuclear dynamics ansatz.^78^ In principle, this choice can be relaxed, and one can utilize various choices of tensor network representation for the expansion coefficients **C**, such as matrix product states or hierarchical Tucker formats, which when employed in the multilayer extension^79,80^ of MCTDH allow for an increase in efficiency for certain problems. However, since the time dependence of the ansatz in eq 9 is entirely within the expansion coefficients, one only needs to calculate the matrix elements at time zero, creating a quite efficient time propagation framework. Note that although we use a simple Hartree product over electronic degrees of freedom, the above ansatz can be straightforwardly extended to have fermionic antisymmetry via treating the CWFs as the spatial component of spin orbitals in Slater determinants.

Hereafter, and for reasons that will be apparent later, we will call eq 9 the static-basis ICWF (or sta-ICWF) ansatz. With this ansatz in hand, we then consider a solution of eq 1 based on the imaginary-time propagation technique,^81^ i.e.
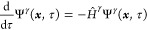
10where
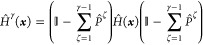
11and *P̂*^ζ^ = Ψ^ζ^Ψ^ζ†^ are projectors used to remove the wave functions Ψ^ζ^ from the Hilbert space spanned by *Ĥ*. The first excited state, for instance, is thus obtained by removing the ground state from the Hilbert space, which makes the first excited state the ground state of the new Hamiltonian.

By introducing the ICWF ansatz of eq 9 into eq 10, we find an equation of motion for the coefficients **C**^γ^ = {*C*_1_^γ^, ..., *C*_*N*_*c*_*M*_^γ^}
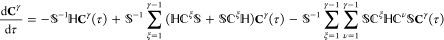
12where , and the matrix elements of  and  are
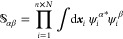
13
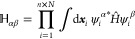
14where again, the α, β indices refer to the index over particle placement and excited CWFs. Obtaining these matrix elements involves a sum over all two-body interactions across each degree of freedom and a sum across one-body operators. In practice,  may be nearly singular, but its inverse can be approximated by the Moore–Penrose pseudo-inverse.

Based on solving the system of equations in eq 12 for **C**^γ^, one already has the ingredients to put forth a time-independent ICWF eigensolver algorithm that will ultimately be used to evaluate the expectation value of generic observables *Ô*(***x***). Given an approximate solution to the eigenfunction Ψ^γ^(***x***), the expectation value of  reads
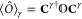
15with the matrix elements of  being given by an analogous expression to eq 14.

### 3.1 Example I: Ground and Excited BOPESs of H_2_

As an illustrative example, we now calculate the BOPESs of a model for the H_2_ molecule. We adopt a model where the motion of all particles is restricted to one spatial dimension, and the center-of-mass motion of the molecule can be separated off.^82,83^ In this model, the relevant coordinates are the internuclear separation, *R*, and the electronic coordinates, *r*_1_ and *r*_2_. The Hamiltonian, written in terms of these coordinates, is
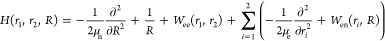
16where for *M* being the proton mass, μ_e_ = *M*/(2*M* + 1) is the reduced electronic mass and μ_n_ = *M*/2 is the reduced nuclear mass. In eq 16, the electron–electron repulsion and the electron–nuclear interaction are represented by soft-Coulomb potentials
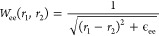
17
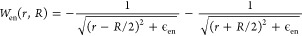
18i.e., the Coulomb singularities are removed by introducing smoothing parameters ϵ_ee_ = 2 and ϵ_en_ = 1. The above model system qualitatively reproduces all important strong-field effects such as multiphoton ionization, above-threshold ionization, or high-harmonic generation.^84−86^ Moreover, it has provided valuable information in the investigation of electron correlation effects.^87−89^

For this model, the BOPESs are defined by the following electronic eigenvalue problem
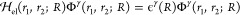
19where , and {Φ^γ^(*r*_1_, *r*_2_; *R*)} are the (complete, orthonormal) set of BO electronic states. A parametric dependence on the nuclear coordinates is denoted by the semicolon in the argument. The BOPESs, ϵ^γ^(*R*), can be calculated using the imaginary-time sta-ICWF method described in eqs 10–14 along with a simplified version of the ansatz in eq 9 that is specialized to this particular case of parametric nuclear dependence. A thorough description of the numerical procedure, as well as the convergence behavior of the sta-ICWF method for this model can be found in Appendix B.1.

**Figure 2:**
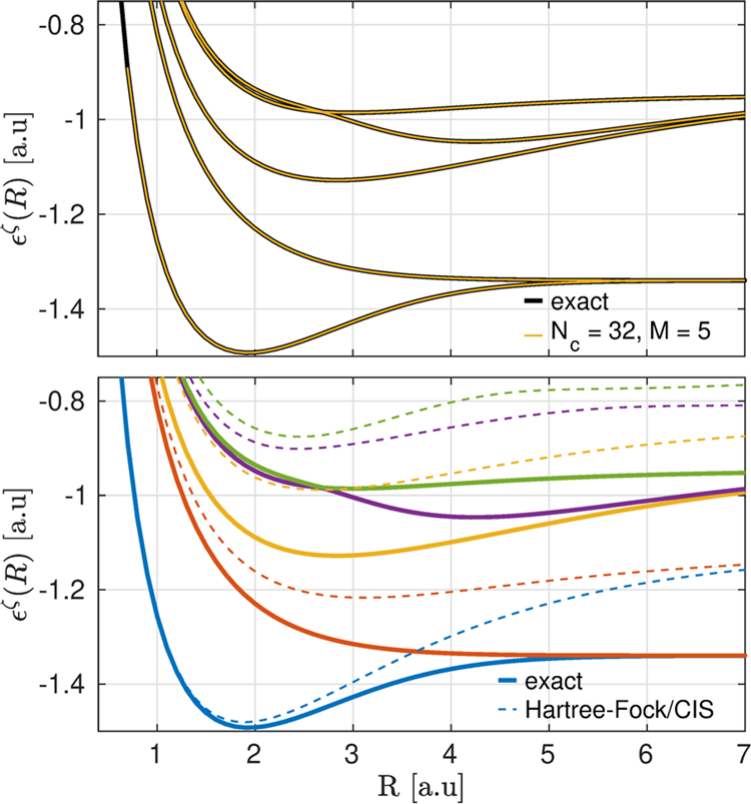
Exact first five BOPESs of the one-dimensional H_2_ model system (solid black lines). sta-ICWF results for (*N*_*c*_, *M*) = (32, 5) are shown in the top panel (solid gold lines). Hartree–Fock and CIS results for the ground-state and excited-state BOPESs, respectively, are shown in the bottom panel (dashed lines) alongside exact results (solid lines) and color-coordinated via calculated excited states.

In Figure 2, we show the first five BOPESs calculated via the sta-ICWF approach using (*N*_*c*_, *M*) = (32, 5). In the top panel, the exact BOPESs are plotted against the sta-ICWF data, overlaid as solid gold lines. The results demonstrate that the sta-ICWF ansatz used in a variational context captures the entire group of the excited BOPES landscape over this energy range. As a point of comparison, in the bottom panel of Figure 2, we also show the result of mean-field-type calculations of the BOPESs for this system. Specifically, we show Hartree–Fock and configuration interaction singles (CIS) data for the ground-state and excited-state BOPESs, respectively, which suffer from well-known inaccuracies in capturing the binding energy and excited-state properties of the system.

## 4 Time-Dependent Properties with Conditional Eigenstates

The sta-ICWF eigensolver described above can be easily extended to describe dynamical properties. For that, we consider the time-dependent Schrödinger equation
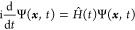
20where Ψ(***x***, *t*) is the electron–nuclear time-dependent wave function, and the Hamiltonian of the system *Ĥ*(*t*) may contain a time-dependent external electromagnetic field.

In practice, we are interested in situations where the initial wave function is the correlated electron–nuclear ground state, i.e., Ψ(***x***, 0) = Ψ^γ=0^(***x***), and some nonequilibrium dynamics is triggered by the action of an external driving field (hereafter, we omit the superscript γ for clarity). We can then decompose the time-dependent many-body wave function as in eq 9 by restricting it to the case of γ = 0. We choose to restrict, for the moment, the time dependence of our ansatz to the expansion coefficients *C*_α_. Although in this formulation the basis remains static, by choosing sufficient excited CWF states, γ > 0 in eq 8, for ***x***_α_ covering some anticipated range of motion for the dynamics, we can expect to capture the support of Ψ(*t*). The equations of motion for *C*_α_ can be obtained either by inserting eq 9 directly into eq 20 or by utilizing the Dirac–Frenkel variational procedure^b^
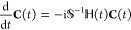
21In eq 21, the matrix elements of  and  are identical to those defined in eqs 13 and 14, with the Hamiltonian’s time dependence coming from any external fields and the wave function decomposed into single-particle CWFs for the nuclear and both electronic degrees of freedom. The values of the coefficients at time *t* = 0, i.e., **C**(0), may be obtained from the imaginary-time sta-ICWF method of eq 12. In this way, the combination of the imaginary-time and real-time sta-ICWF methods yields a “closed-loop” algorithm for the structure and dynamics of molecular systems that does not require explicit BO state information as an input to the method. For the interested reader, a detailed flowchart of the resulting sta-ICWF method can be found in Appendix D.

### 4.1 Example II: Optical Absorption Spectrum of H_2_

Here, we demonstrate an application of the real-time sta-ICWF approach to simulate the optical absorption spectrum for the molecular hydrogen model introduced in Section 3.1. We utilize the “δ-kick” method of Yabana and Bertsch,^90^ where an instantaneous electric field *E*(*t*) = κδ(*t*) with perturbative strength κ ≪ 1 au^–1^ couples to the dipole moment operator μ = *r*_1_ + *r*_2_ and thereby produces an instantaneous excitation of the electronic system to all transition dipole allowed states. The resulting (linear) absorption spectra can then be calculated via the dipole response, Δμ(*t*) = μ(*t*) – μ(0^–^)
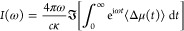
22In practice, due to the finite time propagation, the integrand is also multiplied by a mask function  that smoothly vanishes at the final simulation time *T*_f_.

The system is first prepared in the ground state using the imaginary-time sta-ICWF. See Appendix B.2 for a thorough description of the imaginary-time sta-ICWF method and its use for preparing the ground state of the H_2_ model system. The field-driven dynamics is then generated by applying the kick operator to the relevant degree of freedom. A thorough description of the numerical procedure, as well as the convergence behavior of the sta-ICWF method for this model, can be found in Appendix B.3. The reader can also find a detailed flowchart of the (real and imaginary) sta-ICWF method in Appendix D.

**Figure 3:**
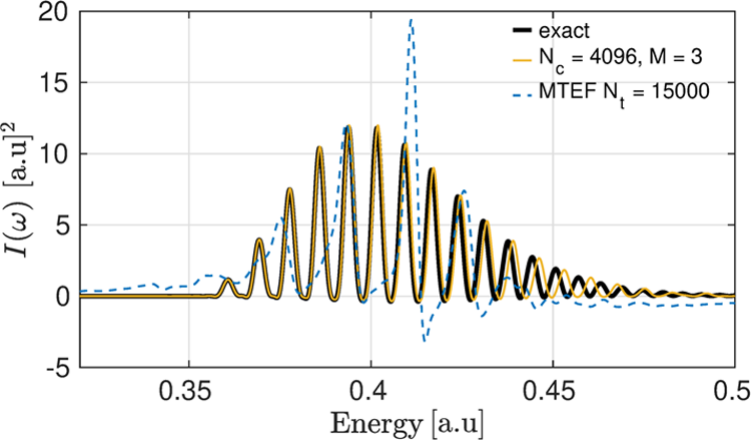
S_2_ ← S_0_ spectra of ICWF-kick (gold) and multitrajectory Ehrenfest δ-kick (MTEF-kick) (blue) compared to the exact peak placement overlaid as a black line, showing that while mean-field theory is unable to capture qualitatively the correct vibronic line shape spacing and intensity, the sta-ICWF approach accurately captures the exact spectrum.

For the H_2_ model, the occupation of excited electronic states and subsequent coupled electron–nuclear dynamics produce a characteristic vibronic peak structure usually explained via the Franck–Condon vertical transition theory. In the top panel of Figure 3, we show vibronic spectra calculated both with sta-ICWF for the absorption from *S*_0_ to *S*_2_ in comparison with the numerically exact results also calculated via the δ-kick approach. For sta-ICWF, we found that *N*_*c*_ = 4096 and *M* = 3 was sufficient to obtain accurate results. The results demonstrate that the sta-ICWF ansatz used in a variational context achieves an accurate vibronic spacing, and furthermore, it not only captures the electron–nuclear correlation inherent to vibronic spectra but also solves the electron–electron subsystem accurately. The deviation from the exact results does grow with increasing energy, although this is ameliorated with increasing *N*_*c*_ and *M*, and can, in principle, be eliminated at large enough values of these parameters (see Appendix B.3).

For comparison, we also show mean-field, semiclassical results for the vibronic spectra. Specifically, we calculated the absorption spectrum with the multitrajectory Ehrenfest δ-kick (MTEF-kick) method,^61^ overlaid as dashed blue lines. The electronic subsystem was solved exactly as a two-particle wave function over the real-space grid for each independent nuclear trajectory. We see that the vibronic spacing calculated with the MTEF-kick approach fails in capturing the correct peak spacing in addition to showing unphysical spectral negativity.

### 4.2 Example III: Laser-Driven Dynamics of H_2_

The present formalism is not restricted to just perturbative fields and can deal with any arbitrary external field. Going beyond the linear response regime, we investigate the effect of strong driving by a few-cycle, ultrafast laser pulse for this same H_2_ model system. The system is first prepared in the ground state using the imaginary-time sta-ICWF, and then the field-driven dynamics is generated by applying an electric field of the form *E*(*t*) = *E*_0_Ω(*t*) sin(ω*t*), with *E*_0_ = 0.005 au and an envelope Ω(*t*) with a duration of 20 optical cycles. The carrier wave frequency ω = 0.403 is tuned to the vertical excitation energy between the ground and second excited BOPESs at the mean nuclear position of the ground-state wave function. A thorough description of the numerical procedure, as well as the convergence behavior of the sta-ICWF method for this model, can be found in Appendix B.4, as well as in Appendix D.

**Figure 4:**
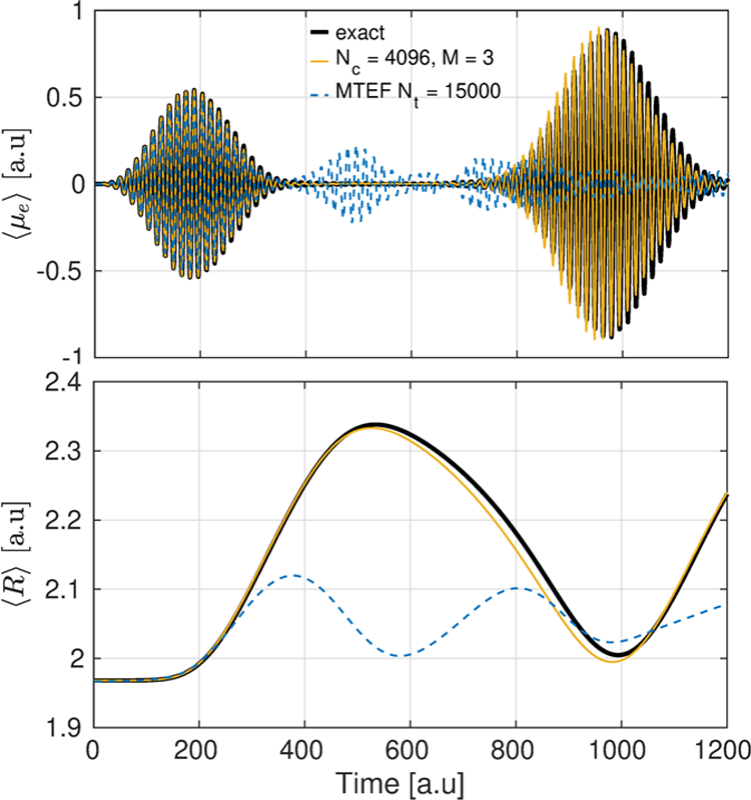
Top panel: evolution of the expectation value of the dipole operator ⟨μ_e_⟩ for the 1D H_2_ model system for *N*_*c*_ = 4096 (from bottom-up) and *M* = 3. Bottom panel: evolution of the expectation value of the nuclear interseparation ⟨*R*⟩ for the 1D H_2_ model system for *N*_*c*_ = 4096 and *M* = 3.

The intense laser pulse creates a coherent superposition of the ground and second excited BO states whereby the bond length of the molecule increases, as shown in the bottom panel of Figure 4. The nuclear wavepacket then eventually returns to the Franck–Condon region, creating the resurgence of the electronic dipole oscillation seen in the top panel of Figure 4. In the MTEF mean-field description of this process, the short-time limit is rather accurately captured, while the subsequent effects of the laser pulse on the nuclear dynamics and the resurgence in the dipole response are not. These results show that the sta-ICWF method is able to capture the electronic correlations inherent to the electronic dipole moment during the initial laser-driven dynamics, as well as the electron–nuclear correlations that arise during the subsequent nonequilibrium dynamics. For this particular problem, we found that (*N*_*c*_, *M*) = (4096, 3) was sufficient to obtain highly accurate results for both the expectation value of the electronic dipole moment (top panel of Figure 4) and the expectation value of the internuclear separation (bottom panel of Figure 4). Further details can be found in Appendix B.4.

## 5 Time-Dependent Conditional Wave Functions

While the sta-ICWF method shows promising performance in the examples studied thus far, it faces the same limitations as any method that relies on a static basis. Perhaps, the most significant aspect can be framed in terms of capturing the full support of the time-dependent wave function, which is exacerbated in cases where the time-dependent state strays far from the span of the static basis. One strategy to address these scenarios would be to incorporate time-dependent conditional wave functions in the ICWF ansatz. Hence, we take advantage of the time-dependent version of the CWF framework introduced in ref (69), which relies on decomposing the exact many-body wave function, Ψ(***x***, *t*), in terms of time-dependent single-particle CWFs of either the electronic or nuclear subsystems as
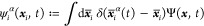
23

Evaluating the time-dependent Schrödinger equation in eq 20 at ***x***_*i*_^α^(*t*), one can show that the CWFs in eq 23 obey the following equations of motion
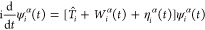
24where *W*_*i*_^α^(***x***_*i*_, *t*) = *W*(***x***_*i*_,***x*®**_*i*_^α^(*t*), *t*), and we remind that *W*(***x***) is the full electron–nuclear interaction potentials that appear in the Hamiltonian of eq 2. In eq 24, η_*i*_^α^(***x***_*i*_, *t*) are time-dependent complex potentials containing kinetic correlations and advective terms, i.e.
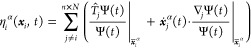
25

As in the time-independent CWF framework, the conditional wave functions in eq 23 represent slices of the full wave function taken along single-particle degrees of freedom of the two disjoint subsets. Each individual CWF constitutes an open quantum system, whose time evolution is nonunitary, due to the complex potentials η_*i*_^α^(***x***_*i*_, *t*), which now include advective terms due to the inherent motion of the trajectories ***x***^α^(*t*), which evolve according to Bohmian (conditional) velocity fields^69^
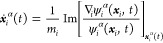
26An exact solution to eq 20 can be then constructed provided we use a sufficiently large number of slices {***x***^α^(*t*)} that explore the full support of |Ψ^γ^(***x***, *t*)|^2^ (in analogy with Figure 1b), i.e.
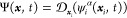
27where the transformations can be found in Appendix A. The one-body equations of motion in eq 24 can be then understood each as a coupled set of nonunitary and nonlinear time-dependent problems.

The derivation of the exact time-dependent CWF mathematical framework corresponds to the transformation of the many-body time-dependent Schrödinger equation to the partially comoving frame in which all coordinates except the *i*th move attached to the electronic and nuclear flows and only the *i*th coordinate is kept in the original inertial frame. Within the new coordinates, the convective motion of all degrees of freedom except for the *i*th coordinate is described by a set of trajectories of infinitesimal fluid elements (Lagrangian trajectories), while the motion of the *i*th degree of freedom is determined by the evolution of the CWFs in a Eulerian frame.^72^ The purpose of this partial time-dependent coordinate transformation is to propagate all trajectories along with the corresponding probability density flow such that they remain localized where the full molecular wave function has a significant amplitude.

### 5.1 Time-Dependent Hermitian Approximation

In general, the effective potentials in eq 25 exhibit discontinuous steps, which could introduce instabilities in a trajectory-based solution of the many-body dynamics based on eq 24. Therefore, in a similar manner to the time-independent case, an approximate solution can be formulated by expanding the kinetic and advective correlation potentials around the conditional coordinates ***x***^α^(*t*), such that
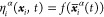
28In this limit, the kinetic and advective correlation potentials only engender a global phase that can be omitted, as expectation values are invariant under such global phase transformations. The resulting propagation scheme is restored to a Hermitian form. That is, eq 24 is approximated as
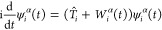
29while the trajectories ***x***^α^(*t*) are constructed according to eq 26.

This approximation to the time-dependent CWF formalism is clearly a major simplification of the full problem, as it recasts the many-body time-dependent Schrödinger equation as a set of independent single-particle equations of motion. Despite the crudeness of the approximation in eq 28, the set of equations of motion in eq 29 has found numerous applications, e.g., in the description of adiabatic and nonadiabatic quantum molecular dynamics^69,71^ and quantum electron transport.^91−95^ In ref (69), for example, results using eq 29 for an exactly solvable model system showed a great degree of accuracy of the time-dependent Hermitian approximation in capturing nonadiabatic dynamics. Alternatively, in ref (71), the set of equations in eq 29 was used to describe the adiabatic double proton transfer for an exactly solvable model porphine, showing great promise in capturing quantum nuclear effects. Regarding the comparison of the time-dependent Hermitian approach in eq 29 with conventional mean-field methods, in ref (92), it was shown that quantum electron transport simulations using eq 29 represent an improvement with respect to time-dependent (Hartree-type) mean-field simulations. Similar conclusions were reported in ref (96), where a simplified semiclassical method based on eq 29 was compared with classical mean-field results.

Methods based on eq 29, however, are known to fail to describe important nonadiabatic processes such as the splitting of the time-dependent reduced nuclear density with influences from different BOPESs.^69^ This type of dynamics has been commonly associated with decoherence effects that neither the Hermitian approximation in eq 28 nor other mean-field methods such as Ehrenfest or Tully’s surface hopping dynamics are able to capture.

## 6 Simulating Far-from-Equilibrium Dynamics with Conditional Wave Functions

In general circumstances where the kinetic and advective correlation potentials are important, we can make use of the simple Hermitian form of the conditional equations of motion in eq 29 to design an efficient many-body wave function propagator. For that, we expand the full electron–nuclear wave function using the ansatz
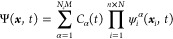
30where the coefficients *C*_α_(*t*) and the CWFs ψ_*i*_^α^(***x***_*i*_, *t*) are initialized using the sta-ICWF method and propagated afterward using the approximated equations of motion in eq 29 along with trajectories obeying eq 26.

The time evolution of the coefficients **C**(*t*) can be then obtained by inserting the ansatz of eq 30 into eq 20
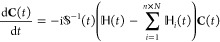
31where the matrix elements of ,  are defined as in eqs 13 and 14, with the time dependence coming from external fields in the Hamiltonian and the time-dependent CWFs, while  are
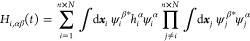
32where *h*_*i*_^α^(*t*) are the Hermitian Hamiltonians in eq 29 and *Ĥ*(*t*) is the full time-dependent Hamiltonian in eq 20.

Obtaining these matrix elements is straightforward, involving a sum across single-body operators in eqs 13 and 32 and all sums of two-body interactions across each degree of freedom in eq 14. Note that any operator involving only a single species, e.g., the kinetic energy, is canceled out, and thus the evolution of **C** is governed exclusively by matrix elements of operators, which either fully (through ) or conditionally (through ) correlate the degrees of freedom.

Equations 26, 29, and 31 define a set of coupled differential equations that hereafter will be referred to as the dynamical ICWF (dyn-ICWF) method. One can then evaluate the expectation value of a generic observable ⟨*Ô*(***x***)⟩ as given in eqs 15 with dyn-ICWF by simply taking into account that ψ_*i*_^α^(*t*) are now time-dependent CWFs.

The above dyn-ICWF method was first put forth in ref (73). At the time of publishing the work in ref (73), however, there was no theory sustaining the construction of the initial conditional wave function basis ψ_*i*_^α^(***x***_*i*_,*t*) without relying on an exact solution of the time-independent Schrödinger equation. That has been the main limitation of the method thus far. Here, instead, we have shown that the imaginary-time sta-ICWF method (derived in Section 3) not only allows us to solve accurately the time-independent Schrödinger equation but also serves as a method to define an optimal set of conditional wave function basis ψ_*i*_^α^(***x***_*i*_,0). Therefore, the dyn-ICWF in combination with imaginary-time sta-ICWF provides a self-consistent approach to describe observables that are relevant to equilibrium, as well as far-from-equilibrium processes. An example combining these two methods will be shown in the example of Section 6.2, where an initial ground state is prepared using imaginary-time sta-ICWF and a later dynamics, triggered by a laser pulse, is described using dyn-ICWF. The interested reader can find a complete flowchart of the combined method in Appendix D.

### 6.1 Example IV: Impact Electron Ionization

The theoretical description of electron scattering remains challenging, as it is a highly correlated problem that generally requires treatment beyond perturbation theory.^97,98^ We here study a model system of electron–hydrogen scattering that can be exactly solved numerically.^99^ In atomic units, the Hamiltonian of this one-dimensional two-electron model system reads
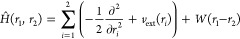
33where
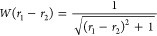
34
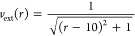
35are, respectively, the soft-Coulomb interaction and the external potential that models the H atom located at *r* = 10 au. The initial interacting wave function is taken to be a spin singlet, with a spatial part
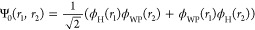
36where ϕ_H_(*r*) is the ground-state hydrogen wave function and ϕ_WP_(*r*) is an incident Gaussian wavepacket
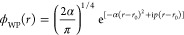
37with α = 0.1 representing an electron at *r* = −10 au, approaching the target atom with a momentum *p*.

The time-resolved picture presents scattering as a fully nonequilibrium problem, where the system starts already in a nonsteady state, and so, the imaginary-time sta-ICWF cannot be applied here to prepare the initial wave function. Instead, we stochastically sample the initial probability density |Ψ_0_(*r*_1_, *r*_2_)|^2^ with *N*_*c*_ trajectories {*r*_1_^α^(0), *r*_2_^α^(0)} that are used to construct CWFs ϕ_1_^α^(*r*_1_, 0) and ϕ_2_^α^(*r*_2_, 0), as defined in eq 23. A thorough description of the numerical procedure, as well as the convergence behavior of the dyn-ICWF method for this model can be found in Appendix C.1. See also Appendix D for a description of the corresponding workflow.

**Figure 5:**
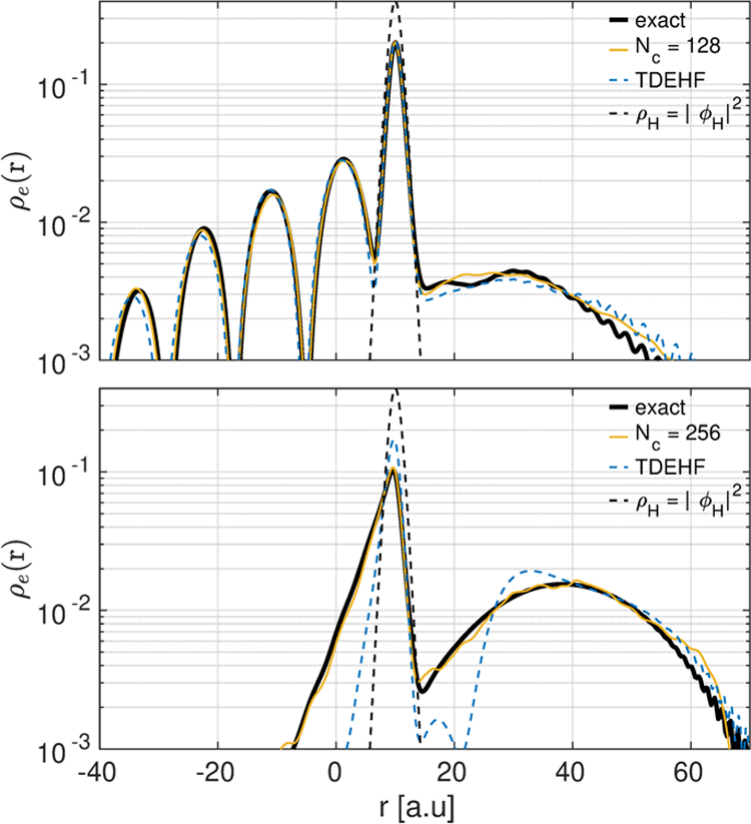
Top panel: reduced electron density at *t* = 1.8 fs for *p* = 0.3 au and *N*_*c*_ = 128. Bottom panel: reduced electron density at *t* = 0.85 fs for *p* = 1.5 au and *N*_*c*_ = 256 and *N*_in_ = 10.

We study the dynamics of the electron–hydrogen scattering by evaluating the time-dependent one-body density, ρ_e_(*r*_1_, *t*) = 2∫|Ψ(*r*_1_, *r*_2_, *t*)|^2^  d*r*_2_, for two different initial momenta, viz., *p* = 0.3 and 1.5 au. For *p* = 0.3 au, the energy is lower than the lowest excitation of the target (which is about ω = 0.4 au) and hence the scattering process is elastic. In this regime, mean-field results (here represented by extended time-dependent Hartree–Fock calculations) and dyn-ICWF results with *N*_*c*_ = 128 results both capture the correct dynamics accurately (see Figure 5). In approaching the target atom with the larger momentum *p* = 1.5 au, the incident wavepacket collides inelastically with the target electron at around 0.24 fs, after which, a part of the wavepacket is transmitted while some is reflected back leaving the target partially ionized. In this regime, the mean-field method fails to describe the transmission process quantitatively and the reflection process even qualitatively due to its inability to capture electron–electron correlation effects. This is in contrast with dyn-ICWF results, which quantitatively capture the correlated dynamics for *N*_*c*_ = 256, although a lower number of CWFs already reproduces qualitatively the dynamics (see Appendix C.1 and Figure 5).

### 6.2 Example V: Laser-Driven Proton-Coupled Electron Transfer

We now show dyn-ICWF results for a prototypical photoinduced proton-coupled electron transfer reaction, using the Shin–Metiu model.^100^ The system comprises donor and acceptor ions, which are fixed at a distance *L* = 19.0*a*_0_, and a proton and an electron that are free to move in one dimension along the line connecting the donor–acceptor complex. Based on the parameter regime chosen, this model can give rise to a number of challenging situations where electron–nuclear correlations play a crucial role in the dynamics.

The total Hamiltonian for the system is
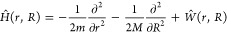
38where *m* is the electron mass, and *M* is the proton mass. The coordinates of the electron and the mobile ion are measured from the center of the two fixed ions and are labeled *r* and *R*, respectively. The full electron–nuclear potential reads
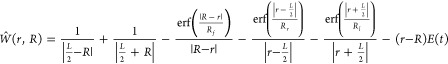
39where erf() is the error function. The parameter regime studied for this model (*R_f_* = 5*a*_0_, *R*_*l*_ = 4*a*_0_, and *R*_*r*_ = 3.1*a*_0_) is chosen such that the ground-state BOPES, ϵ_BO_^1^, is strongly coupled to the first excited adiabatic state, ϵ_BO_^2^, around the mean nuclear equilibrium position *R*_eq_ = −2*a*_0_. The coupling to the rest of the BOPESs is negligible.

**Figure 6:**
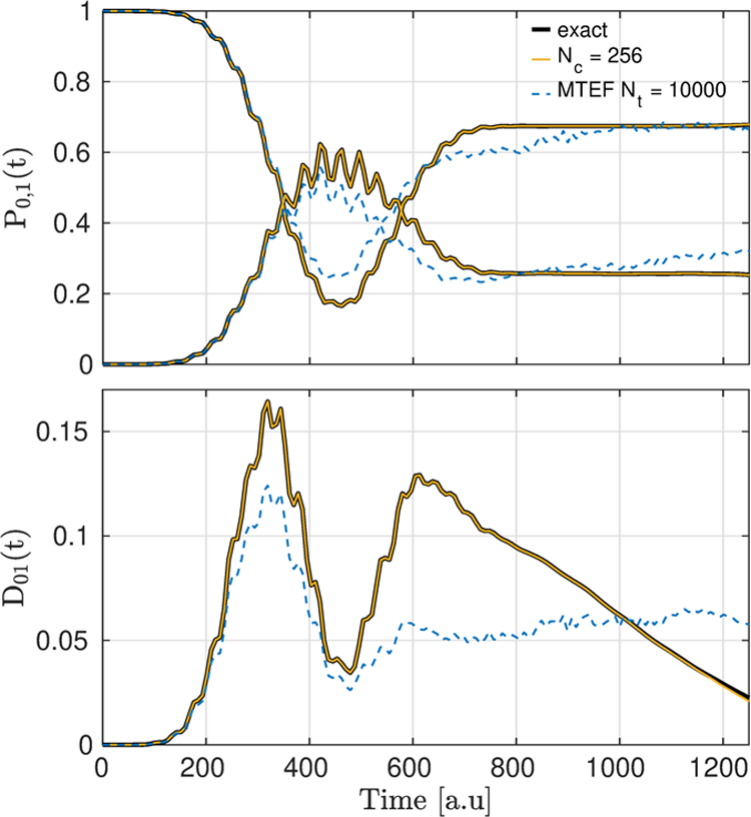
Top panel: population dynamics of the first two adiabatic electronic states *P*_0,1_(*t*). Solid black lines correspond to exact numerical results. Solid blue and red lines correspond to dyn-ICWF results with (*N*_*c*_, *M*) = (256, 1) for the ground and first excited adiabatic populations, respectively. Dashed blue and red lines correspond to mean-field MTEF results. Bottom panel: decoherence dynamics between the ground state and first excited adiabatic electronic states, i.e., *D*_01_. Solid black lines correspond to exact results. The solid blue line corresponds to dyn-ICWF results with (*N*_*c*_, *M*) = (256, 1). The dashed blue line corresponds to mean-field MTEF results.

We set the system to be initially in the full electron–nuclear ground state obtained from the imaginary-time propagation method described above, i.e., Ψ(*r*, *R*, 0) = Ψ^0^(*r*, *R*) (the interested reader can find a general workflow of the simulation in Appendix D). We then apply an external strong electric field, *E*(*t*) = *E*_0_Ω(*t*) sin(ω *t*), with *E*_0_ = 0.006 au, Ω(*t*) = sin(π*t*/20)^2^, and ω = ϵ_BO_^1^(*R*_eq_) – ϵ_BO_^0^(*R*_eq_). The external field induces a dynamics that involves a passage through an avoided crossing between the first two BOPESs, with further crossings occurring at later times as the system evolves. When the system passes through the nonadiabatic coupling region, the electron transfers probability between the ground state and the first excited state. This is shown in the top panel of Figure 6, where we monitor the BO electronic state populations *P*_*n*_(*t*) (whose definition can be found in Appendix C.2). As a result of the electronic transition, the reduced nuclear density changes shape by splitting into two parts representing influences from both ground- and excited-state BOPESs. This can be seen in the bottom panel of Figure 6, where, as a measure of decoherence, we use the indicator *D*_*nm*_(*t*) (whose definition can be found in Appendix C.2). As nonadiabatic transitions occur, the system builds up a degree of coherence that subsequently decays as the system evolves away from the coupling region.

As shown in Figure 6, the dyn-ICWF method reaches quantitative accuracy for (*N*_*c*_, *M*) = (256, 1) and vastly outperforms the multitrajectory Ehrenfest mean-field method in describing both the adiabatic populations and the decoherence measure. More specifically, while both the dyn-ICWF method and MTEF dynamics correctly capture the exact adiabatic population dynamics at short times, the latter breaks down at long times as it fails to capture the qualitative structure of the time-evolving indicator of decoherence. Noticeably, all of these aspects of this problem are qualitatively well described by the dyn-ICWF method using only (*N*_*c*_, *M*) = (16, 1) (these results can be found in Appendix C.2).

### 6.3 Example VI: Interference Effects Near a Molecular Conical Intersection

We next study dynamics around conical intersections (CIs) using a minimal generalization of the above Shin–Metiu model first proposed by Gross and co-workers^101^ and extended further by Schaupp and Engel.^102^ The model consists of a quantized electron and proton that can move in two Cartesian directions, along with two fixed “classical” protons, ***R***_1_, ***R***_2_. A CI occurs in this model when (treating the quantized proton as a BO parameter) the protons are in a *D*_3*h*_ geometry. The potential energy is
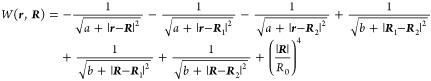
40and we use the parameter values *a* = 0.5, *b* = 10, *R*_0_ = 1.5, ***R***_1_ = (−0.4√3, 1.2), and ***R***_2_ = (0.4√3, 1.2).

**Figure 7:**
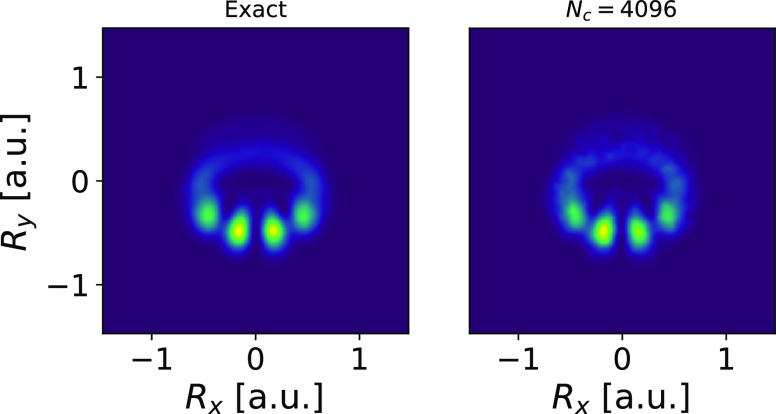
Exact and dyn-ICWF reduced nuclear density showing the interference pattern after having traversed the conical intersection at the origin.

**Figure 14:**
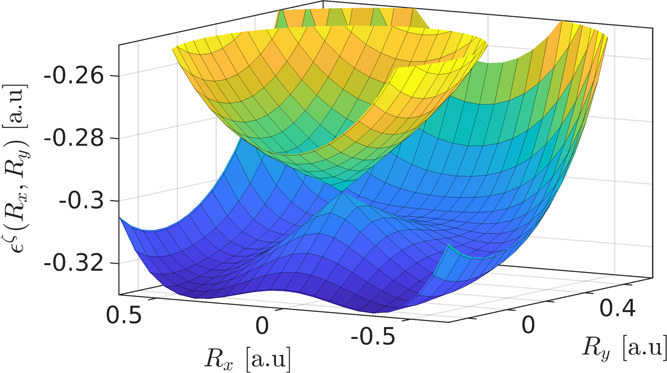
BOPESs for the first two excited states with electronic quantum numbers ζ = 1 (lower surface) and ζ = 2. As mentioned in the main text, the initial nuclear state is initialized as a Gaussian centered at ***R*** = (0, 0.4) on the lower surface.

We initialize the total system wave function as a direct product of the first excited electronic BO state and a nuclear Gaussian state centered at ***R***_c_ = (0, 0.4) with standard deviation σ^2^ = 5. For this placement of ***R***_1_, ***R***_2_, the CI occurs at the origin and, in the BO picture, the initial nuclear wavepacket “falls toward” the CI (see Figure 14 in Appendix C.3). In this picture, the nuclear motion occurs on a single BOPES and the two portions of the nuclear wavepacket around the CI (i.e., the clockwise and anticlockwise components) cause an interference pattern to develop when they do recollide (see Figure 7).

While the interference pattern described in Figure 7 can be understood as the adiabatic circular motion around the position of a conical intersection, it is important to emphasize that the concept of CI makes sense only when the adiabatic picture, i.e., the Born–Huang basis expansion, is used to represent the molecular wave function. However, any observable effect that can be explained on the adiabatic basis must arise also in any other picture such as the diabatic picture or the full real-space grid picture used by the dyn-ICWF method. Therefore, while not depending on the BO picture (beyond defining the initial state), the dyn-ICWF method is able to capture the correct CI curvature effects, as well as any interference pattern that forms in the fully reduced nuclear density
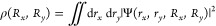
41See Appendices C.3 and D for further details on the dyn-ICWF calculation.

## 7 Conclusions

In this work, we have introduced an exact mathematical framework that avoids the standard separation between electrons and nuclei and hence enables a unified treatment of molecular structure and nonadiabatic dynamics without relying on the construction and fit of Born–Oppenheimer potential-energy surfaces and the explicit computation of nonadiabatic couplings.

We have introduced a time-independent conditional wave function theory, which is an exact decomposition and recasting of the static many-body problem that yields a set of single-particle conditional eigenstates. Based on the imaginary-time propagation of a stochastic ansatz made of approximated conditional eigenstates, the resulting method, called sta-ICWF, is able to accurately capture electron–electron correlations intrinsic to molecular structure. A real-time counterpart of the above method has been also derived following the Dirac–Frenkel variational procedure, and its combination with the imaginary-time version yields an accurate method for solving out-of-equilibrium properties of molecular systems where nonadiabatic electron–nuclear correlations are important. This has been shown by reproducing the exact structural, linear response, and nonperturbatively driven response properties of an exactly solvable one-dimensional H_2_ model system that standard mean-field theories fail to describe.

We have also considered a broader class of conditional wave functions that was formally introduced through time-dependent conditional wave function theory, yielding a set of coupled single-particle equations of motion. An approximated set of these time-dependent conditional wave functions are utilized as time-dependent basis of a stochastic wave function ansatz that is meant to describe observables that are relevant to far-from-equilibrium processes. The resulting propagation technique (called dyn-ICWF) in combination with sta-ICWF provides a fully self-consistent approach and, moreover, the method achieves quantitative accuracy for situations in which mean-field theory drastically fails to capture qualitative aspects of the combined electron–nuclear dynamics.

The sta- and dyn-ICWF methods are wave function-based approaches. Therefore, while the simple sum-of-product forms that we have employed for our ansatz in eqs 9 and 30 can be made more efficient, by introducing a tensor network representation for the expansion coefficients such as matrix product states or hierarchical Tucker formats, for example, an exponential scaling with respect to the number of correlated degrees of freedom is expected unless approximations are introduced. That being said, we want to emphasize that the ICWF method is fundamentally different from wave function methods that rely on the Born–Huang expansion of the molecular wave function. Alternatively, the ICWF method describes electronic and nuclear degrees of freedom on the same mathematical footing, viz., the real-space grid picture. It is this particular trait that makes the ICWF an original starting point for developing novel, unexplored, approximations that could eventually yield a significant computational advantage compared to methods that rely on the Born–Huang expansion.

Importantly, the conditional decomposition holds for an arbitrary number of subsets (up to the total number of degrees of freedom in the system) and applies to both fermionic and bosonic many-body interacting systems. Our developments thus provide a general framework to approach the many-body problem in and out of equilibrium for a large variety of contexts. For example, using conditional wave functions in a form compatible with time-dependent density functional theory in connection with alternative tensor network decompositions or in combination with classical/semiclassical limits for specified degrees of freedom are particularly appealing routes to follow, and work in this direction is already in progress.^103^ Furthermore, the extension to periodic systems is currently under investigation and should allow the ab initio description of driven electron–lattice dynamics such as, for example, laser-driven heating and thermalization,^104−109^ correlated lattice dynamics,^110−112^ and phase transitions.^113−115^

## Figures and Tables

**Figure 8:**
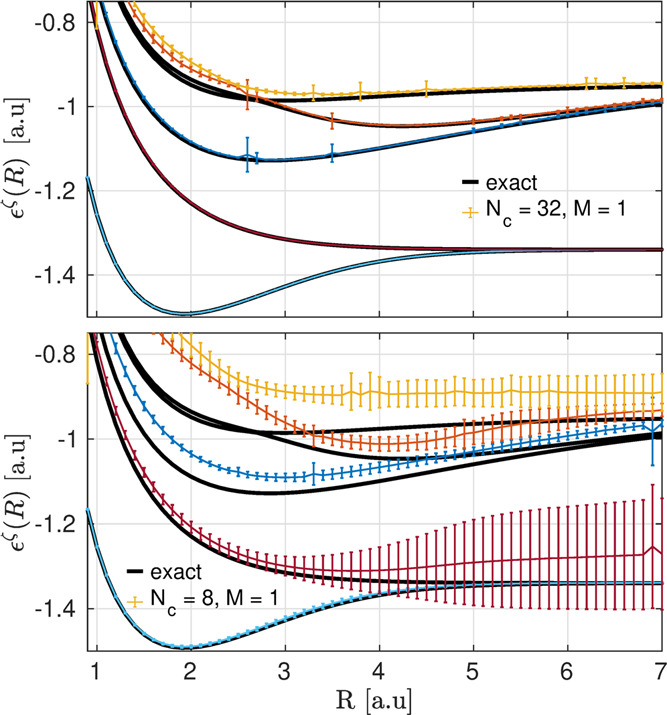
First five BOPESs reproduced with the sta-ICWF method for (*N*_*c*_, *M*) = (8, 1) (bottom panel) and (*N*_*c*_, *M*) = (32, 1) (top panel). These data are presented alongside (standard deviation) error bars.

**Figure 9:**
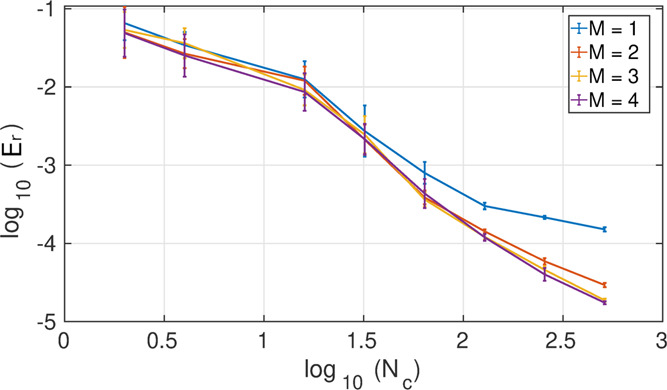
(Left) Logarithm of the mean relative energy error *E*_er_ as a function of the logarithm of the number of sampling points *N*_*c*_ and for different number of excited CWFs *M* = {1, 2, 3, 4}. Error bars represent the standard deviation of the relative error.

**Figure 10:**
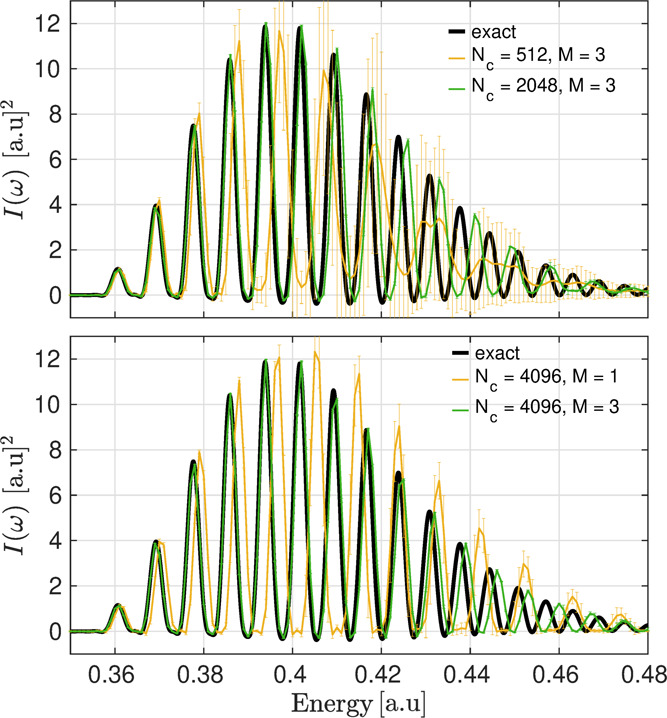
H_2_ spectrum for ICWF-Kick with different number of sampling points and excited CWFs. Top panel: (*N*_*c*_, *M*) = (512, 3) and (*N*_*c*_, *M*) = (2048, 3). Bottom panel: (*N*_*c*_, *M*) = (4096, 1) and (*N*_*c*_, *M*) = (4096, 3). The results are presented alongside (standard deviation) error bars.

**Figure 11:**
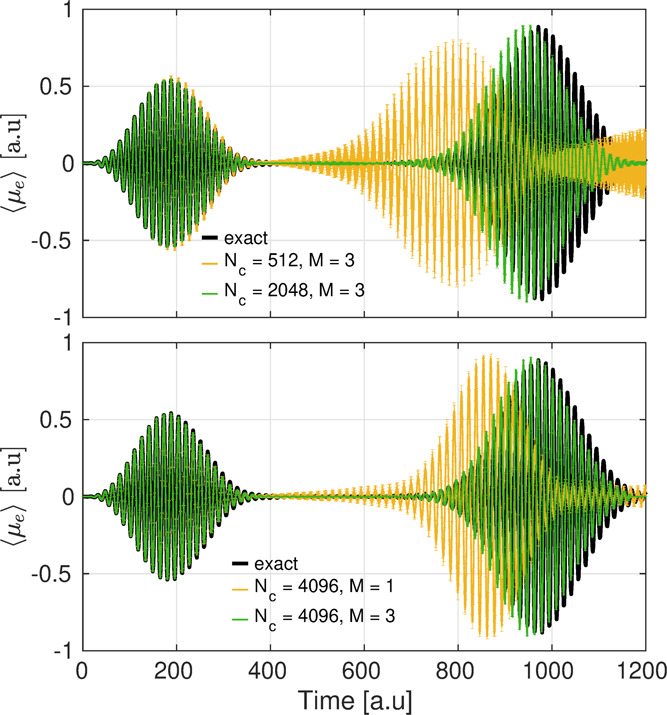
Evolution of the expectation value of the dipole operator ⟨μ_e_⟩ for the 1D H_2_ model system for a number of conditional basis configurations. Top panel: (*N*_*c*_, *M*) = (512, 3), (*N*_*c*_, *M*) = (4096, 3). Bottom panel: (*N*_*c*_, *M*) = (4096, 1) and (*N*_*c*_, *M*) = (4096, 3). These data are presented along with (standard deviation) error bars.

**Figure 12:**
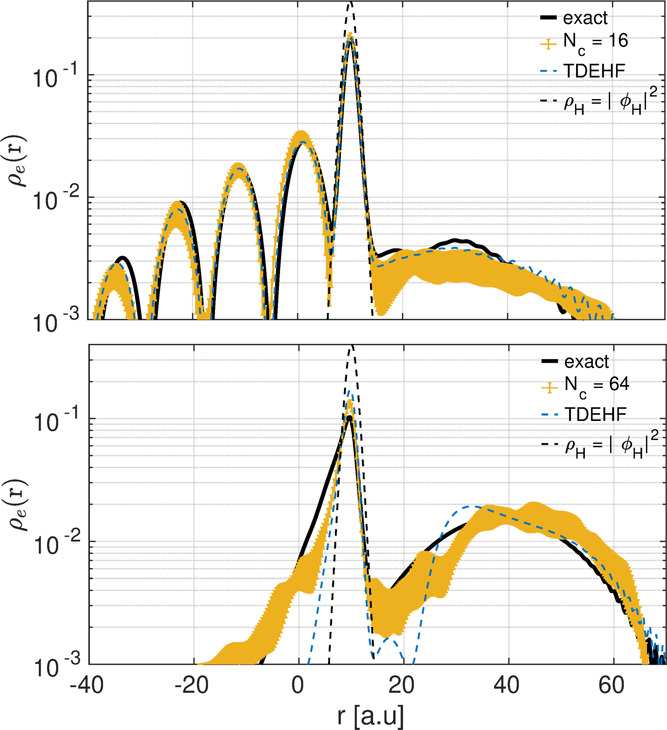
Top panel: reduced electron density at *t* = 1.8 fs for *p* = 0.3 au and (*N*_*c*_, *M*) = (16, 1). Bottom panel: reduced electron density at *t* = 0.85 fs for *p* = 1.5 au and (*N*_*c*_, *M*) = (64, 1).

**Figure 13:**
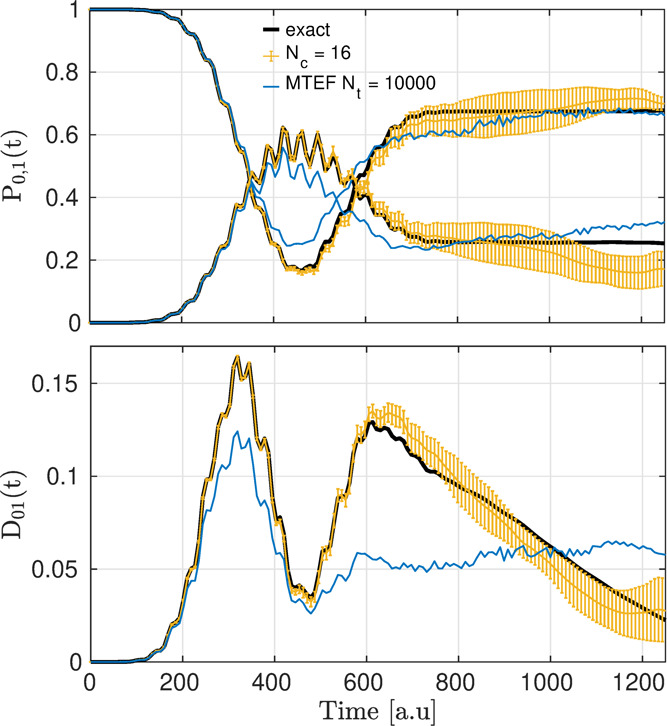
Top panel: population dynamics of the first two adiabatic electronic states *P*_0,1_(*t*). Solid black lines correspond to the exact numerical results. Solid blue and red lines correspond to dyn-ICWF results with (*N*_*c*_, *M*) = (16, 1) for the ground and first excited adiabatic populations, respectively. Bottom panel: decoherence dynamics between the ground state and first excited adiabatic electronic states, i.e., *D*_01_. Solid black lines correspond to exact results. The solid blue line corresponds to dyn-ICWF results with (*N*_*c*_, *M*) = (16, 1).

**Figure 15:**
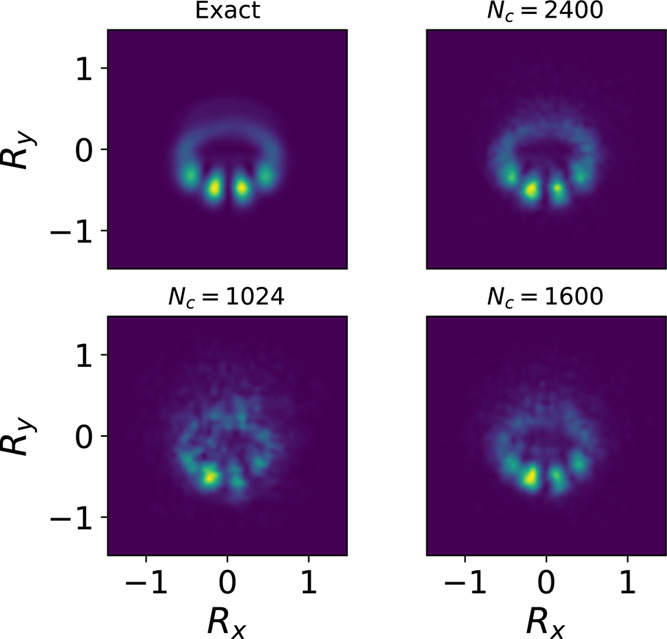
Convergence of the interference pattern arising from the CI with respect to the number of basis elements, *N*_*c*_. The computational time for each fourth-order Runge–Kutta time step scales as  for *a* = 1.59 ± 0.06.

**Figure 16:**
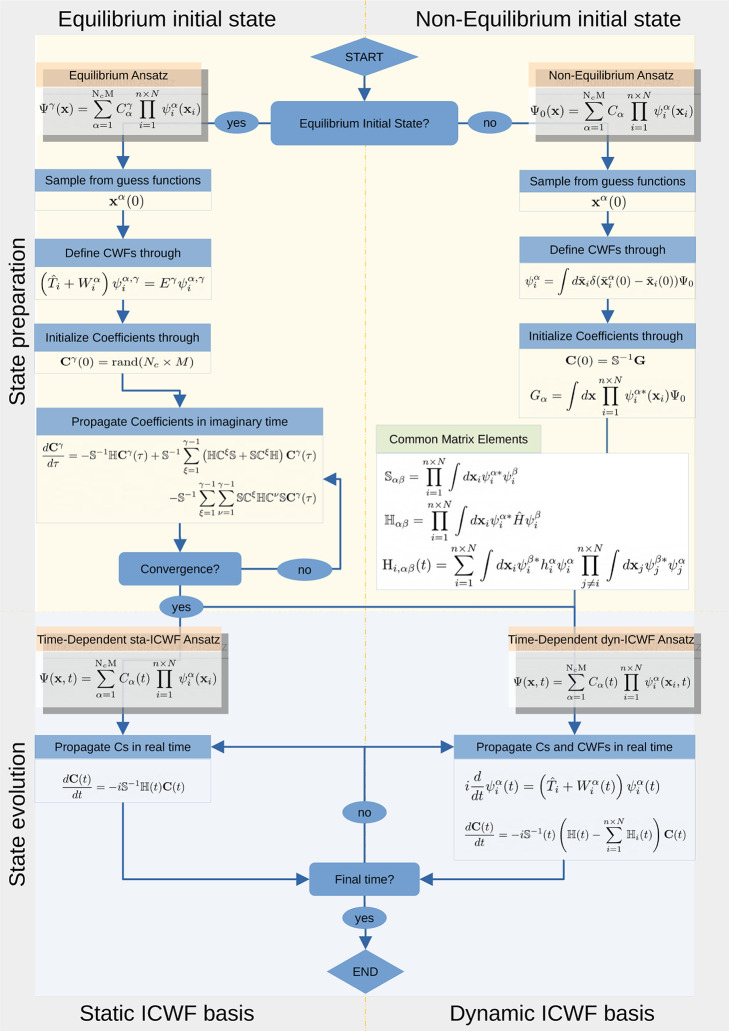
Flowchart of the ICWF method. The flowchart illustrates all possible situations of interest, viz., state preparation using either imaginary-time sta-ICWF for equilibrium states (top-left) or direct matrix inversion for nonequilibrium states (top right), and state propagation using real-time sta-ICWF (bottom left) or dyn-ICWF (bottom right).

## Appendix

### A Definition of the “Reassembling” Transformation of Equation 6

Here, we consider a reconstruction of the full wave function Ψ^γ^(***x***) from conditional wave functions defined as in eq 3 of the main text, i.e.

42

Here, the index α ∈ {1, 2, ..., *N*_*c*_} denotes the particular conditional slice, and ***x*®**_*i*_ = (***x***_1_, ..., ***x***_*i*–1_, ***x***_*i*+1_, ..., ***x***_*n*×*N*_) are the coordinates of all degrees of the system except ***x***_*i*_. Similarly, ***x*®**_*i*_^α^ = (***x***_1_^α^, ..., ***x***_*i*–1_^α^, ***x***_*i*+1_^α^, ..., ***x***_*n*×*N*_^α^) are the position of all system’s degrees of freedom except ***x***_*i*_.

Assuming that the conditional sampling points, ***x*®**_*i*_^α^, are distributed according to a normalized distribution , one can approximately reconstruct the full wave function based on the interpolation with a Gaussian function *G*^σ^(***x*®**_*i*_) with a given width σ as

43

In this way, the full wave function is reconstructed as a Gaussian weighted average: in the numerator of eq 43, the contribution from each conditional slice α is weighted with a Gaussian distribution, and it becomes larger if the evaluated point, ***x*®**, is closer to the sampling point ***x*®**^α^. To compensate the nonuniform sampling distribution contribution, the interpolation weight is divided by the distribution function . In addition, the denominator of eq 43 ensures normalization of the interpolation weight.

By considering a dense sampling (*N*_*c*_ → ∞), the reconstructed wave function of eq 43 can be rewritten as

44

and substituting eq 42 into eq 44, one obtains

45

where ***x*®**′ = (***x***_1_^′^, ..., ***x***_*i*–1_^′^, ***x***_*i*_, ***x***_*i*+1_^′^, ..., ***x***_*n*×*N*_^′^). Therefore, for a dense sampling, Ψ_*N*_*c*_,σ_^Rec,γ^(***x***) can be understood as the convolution of the full wave function Ψ(***x***) and the Gaussian weight *G*^σ^(***x*®**_*i*_). Furthermore, in the narrow Gaussian width limit, (σ → 0), *G*^σ^(***x*®**_*i*_) can be treated as a Dirac δ function and hence eq 45 can be written as

46

In conclusion, one can exactly reconstruct the full electron–nuclear wave function in terms of conditional wave functions using the reassembling operator  defined as

47

## B Convergence of the Real- and Imaginary-Time Versions of the sta-ICWF Method

In this section, we discuss the convergence of the imaginary- and real-time sta-ICWF methods for the examples in Sections 3.1, 4.1, and 4.2. For that, we first notice that, due to the stochastic nature of the sta-ICWF method, given a set of sampling points *N*_*c*_ and their conditional eigenstates *M*, we may also consider a number *N*_in_ of different sets of *N*_*c*_ sampled points and their associated *M* conditional eigenstates. This can be accounted for by rewriting the expectation value of eq 15 as

48

The dispersion of ⟨*O̅*(*t*)⟩ with respect to *N*_in_ is then quantified through its standard deviation, i.e.

49

### B.1 Ground and Excited BOPESs of H_2_

We discuss here the convergence of the imaginary-time version of the sta-ICWF method in capturing the ground-state and excited-state BOPESs for the H_2_ model system introduced in Section 3.1. Finding the BOPESs for this particular model is equivalent to solving eq 19 using the imaginary-time evolution technique

50

where {Φ^γ^(*r*_1_, *r*_2_; *R*)} are the (complete, orthonormal) set of BO electronic states, and we have defined *Ĥ*_el_^ζ^ as

51

where *P̂*^ξ^ = Φ^ξ^Φ^ξ†^ and .

The BO electronic states, Φ^γ^(*r*_1_, *r*_2_; *R*), are then expanded in terms of CWFs with the following simplified version of the ansatz in eq 9 that is specialized to the particular case of parametric nuclear dependence

52

Slicing points (*r*_1_^α^, *r*_2_^α^) are generated by sampling from reduced one-body electronic densities, which in this case are simply chosen to be Gaussian functions ρ_e_(*r*_*i*_) = *A* e^–*r*_*i*_^2^/10^. The conditional eigenstates ϕ_*i*_^α,ν^(*r*_*i*_; *R*), for ν ∈ {1, ..., *M*} are then evaluated on each slice using the Hermitian approximation, i.e.

53

where *W*_*i*_^α^(*r*_*i*_, *R*) = *W*_ee_(*r*_*i*_,*r̅*_*i*_^α^) + *W*_en_(*r*_*i*_, *R*). The coefficient vector **C**^γ^ is randomly initialized and then propagated in imaginary time until the target state is reached according to eq 12 of the main text, with *Ĥ* being substituted with .

To achieve converged results, a grid (0, 9] au for the internuclear separation with 181 grid points is chosen for the nuclear degrees of freedom. For the electron coordinates, the grid covers the interval [−35, +35] au with 200 grid points. The fourth-order Runge–Kutta integration method was used to propagate the imaginary-time sta-ICWF equation of motion (i.e., eq 12) with a time-step dτ = 0.01 au, and the Moore–Penrose pseudo-inversion method with a tolerance of 10^–8^ was used to approximate the numerical inversion of the overlap matrix in eq 13. Importantly, the matrices  and  of eqs 12 and 14 need only be constructed at the initial time, requiring only the repeated multiplication of an *N*_*c*_ × M vector by an *N*_c_^2^ × *M*^2^ matrix for the imaginary-time propagation.

In Figure 8, we show sta-ICWF results for the first five BOPESs for two different sets of parameters: (*N*_*c*_, *M*) = (32, 1) (top panel) and (*N*_*c*_, *M*) = (8, 1) (bottom panel). The sta-ICWF data are presented alongside (standard deviation) error bars defined in eq 49. Noticeably, even for *M* = 1 (i.e., when only ground-state conditional eigenstates are used in the expansion of eq 52), the results in Figure 8 demonstrate the convergence of the imaginary-time sta-ICWF method to the exact BOPESs. For a large enough number of sampling points and excited CWFs, viz., (*N*_*c*_, *M*) ≳ (32, 5), the sta-ICWF results are fully converged to the exact BOPESs and the associated error bars become negligible due to the completeness of the CWF basis.

### B.2 Ground State of H_2_

We investigate here the ground-state energy for the model H_2_ introduced in Section 3.1, as well as the convergence behavior of the imaginary-time version of the sta-ICWF method in capturing it. We aim to solve eq 10, which for this particular model system reduces to

54

where *Ĥ* is the Hamiltonian in eq 16. For that, we choose the conditional eigenstate basis by sampling *N*_*c*_ points (*r*_1_^α^, *r*_2_^α^, *R*^α^) from guesses to the reduced electronic and nuclear densities ρ_e_(*r*_*i*_) = *A*_e_ e^–*r*_*i*_^2^/10^ and ρ_n_(*R*) = *A*_n_ e^–(*R*–2)^2^^, respectively. Starting from the full H_2_ Hamiltonian of eq 16, these positions are then used to construct and diagonalize the Hermitian Hamiltonians in eq 8. In this way, we obtain 3 × *N*_*c*_ × M conditional eigenstates {ϕ_1_^α,ζ^(*r*_1_), ϕ_2_^α,ζ^(*r*_2_), χ^α,ζ^(*R*)}. This allows us to expand the full ground-state wave function as

55

Given a random initialization of the coefficient vector **C**, we then evolve it in imaginary time according to eq 12 and the matrix elements of eqs 13 and 14. To achieve converged results, a grid (0, 9] au for the internuclear separation with 181 grid points is chosen for the nuclear degrees of freedom. For the electron coordinates, the grid covers the interval [−35, +35] au with 200 grid points. The fourth-order Runge–Kutta algorithm with a tolerance of 10^–8^ was used to propagate the imaginary-time sta-ICWF equations of motion with a time-step dτ = 0.01 au, and the Moore–Penrose pseudo-inversion method was used to approximate the numerical inversion of the overlap matrix in eq 13. Importantly, the matrices  and  of eq 12 need only be constructed at the initial time.

From the exact symmetric ground-state wave function, we found an equilibrium separation of ⟨*R*⟩ = 2.2 au and the ground-state energy is *E*_0_ = −1.4843 au We then define the relative error of the sta-ICWF calculation with respect to the exact calculation as *E*_r_ = |⟨*H̅*⟩_0_ – *E*_0_|/|*E*_0_|, where

56

and Ψ^0^ has been defined in terms of CWFs in eq 55.

The error *E*_r_ is presented in Figure 9 as a function of the number of sampling points and for a different number of excited conditional eigenstates, i.e., (*N*_*c*_, *M*). Error bars represent the standard deviation Δ*H̅*_0_ defined in eq 49 for a number of different initial sampling points. Due to the variational nature of the method, the relative error decreases with an increasing number of sampling points *N_c_*. Noticeably, even for *M* = 1 (i.e., when only ground-state conditional eigenstates are used in the expansion of eq 9), the results in Figure 9 demonstrate the convergence of the imaginary-time sta-ICWF method to the exact ground state. The convergence process is accelerated though as we allow a number of excited conditional eigenstates (i.e., *M* > 1) to participate in the ansatz. For a large enough number of basis elements *N*_*c*_ × *M*, the CWF bases become a complete basis of the problem. This is independent of the initial distribution of sampling points and hence the associated error bars vanish for large enough values of *N*_*c*_ × *M*.

### B.3 Optical Absorption Spectrum of H_2_

We discuss here the convergence of the real-time version of the sta-ICWF method in capturing the optical absorption spectrum of the H_2_ model system introduced in Section 3.1. The simulation starts with the preparation of the ground-state coefficients **C**(0) using the imaginary-time version of the sta-ICWF method described in Appendix B.2. The relevant degree of freedom of the kick operator is then applied to each CWF, the Hamiltonian and inverse overlap matrices of eqs 13 and 14 are reconstructed, and **C** is propagated to the desired time according to eq 21. A kick strength of κ = 10^–4^ au^–1^ was sufficient to generate the kick spectra within the linear response regime, and a total propagation time of *T*_f_ = 1500 au was used to generate the spectra, alongside the mask function .

A grid [−35, +35] au with 200 grid points was chosen for the electronic coordinates. The fourth-order Runge–Kutta algorithm was used to propagate the imaginary-time sta-ICWF equations of motion with a time-step d*t* = 0.01 au, and the Moore–Penrose pseudo-inversion method with a tolerance of 10^–8^ was used to approximate the numerical inversion of the overlap matrix in eq 13. Again, the matrices  and  of eq 12 need only be constructed at the initial time.

In Figure 10, we show convergence results for sta-ICWF calculations of the optical linear absorption spectra (eq 22) for four different sets of parameters: (*N*_*c*_, *M*) = (512, 3), (*N*_*c*_, *M*) = (2048, 3) (top panel), and (*N*_*c*_, *M*) = (4096, 1) and (*N*_*c*_, *M*) = (4096, 3) (bottom panel). In all of these cases, we considered a number of different initial sampling points, which have been used to calculate the associated (standard deviation) error bars as in eq 49. As the number of conditional eigenstate basis elements in the ansatz expansion of eq 55 increases, the variational nature of the method ensures convergence to the exact linear absorption line shape. Similarly, the error bars shrink as the number of conditional eigenstates in the basis *N*_*c*_ × M allows us to span the relevant part of the Hilbert space.

### B.4 Laser-Driven Dynamics of H_2_

We discuss here the convergence of the real-time version of the sta-ICWF method in capturing the laser-driven dynamics of the H_2_ model system introduced in Section 3.1. As explained in Section 4.2 of the main text, the system is first prepared in the ground state using the imaginary-time sta-ICWF as explained in Appendix B.2, and then the field-driven dynamics is generated by applying an electric field of the form *E*(*t*) = *E*_0_Ω(*t*) sin(ω*t*), with *E*_0_ = 0.005 au and an envelope Ω(*t*) with a duration of 20 optical cycles. The carrier wave frequency ω = 0.403 is tuned to the vertical excitation between the ground BO state and second excited electronic surface.

For the dynamics we used, a grid (0, 9] au for the internuclear separation with 181 grid points is chosen for the nuclear degrees of freedom. For the electron coordinates, the grid covers the interval [−35, +35] au with 200 grid points. The fourth-order Runge–Kutta algorithm was used to propagate the sta-ICWF equation of motion in eq 21 with a time-step d*t* = 0.01 au, and the Moore–Penrose pseudo-inversion method with a tolerance of 10^–8^ was used to approximate the numerical inversion of the overlap matrix in eq 13.

In Figure 11, we show convergence results for the real-time sta-ICWF calculation of the electronic dipole moment ⟨μ̂_e_⟩. We considered four different sta-ICWF configurations, viz., (*N*_*c*_, *M*) = (512, 3), (*N*_*c*_, *M*) = (4096, 3) (in the top panel), and (*N*_*c*_, *M*) = (4096, 1) and (*N*_*c*_, *M*) = (4096, 3) (in the bottom panel). As the number of CWFs in the ansatz expansion of eq 55 increases, the variational nature of the method ensures convergence to the exact dynamics. The deviation from the exact results does grow with increasing time lapse, although this is ameliorated with increasing either *N*_*c*_ and/or *M* and can, in principle, be eliminated at large enough values of these parameters. Similarly, the error bars become negligible when the CWF bases expand the full support of the Hilbert space explored during the dynamics. This happens for (*N*_*c*_, *M*) ≳ (4096, 3).

## C Convergence of the dyn-ICWF Method

In this section, we discuss the convergence behavior of the dyn-ICWF method for the examples of Sections 6.1–6.3. As it happened for the sta-ICWF method, the stochastic nature of the dyn-ICWF method allows us to consider a number *N*_in_ of different initial sampling points for a given set of parameters (*N*_*c*_, *M*). This is taken into account by writing expectation values as in eq 48 and its standard deviation as in eq 49.

### C.1 Impact Electron Ionization

We discuss here the convergence behavior of the dyn-ICWF method in capturing the laser-driven proton-coupled electron transfer described in Section 6.1.

The time-resolved picture presents scattering as a fully nonequilibrium problem, where the system starts already in a nonsteady state, and so, the imaginary-time sta-ICWF cannot be applied here to prepare the initial wave function. Instead, we stochastically sample the initial probability density |Ψ_0_(*r*_1_, *r*_2_)|^2^ with *N*_*c*_ trajectories {*r*_1_^α^(0), *r*_2_^α^(0)} that are used to construct CWFs ϕ_1_^α^(*r*_1_, 0) and ϕ_2_^α^(*r*_2_, 0), as defined in eq 23. These CWFs are then used to construct the ansatz in eq 30, i.e.

57

with an initial **C** vector that is obtained using

58

where **G** is the vector containing the overlap between the initial wave function and the CWFs, i.e.

59

Given **C**(0), and ϕ_1_^α^(*r*_1_, 0) and ϕ_2_^α^(*r*_2_, 0) for an ensemble of sampling points {*r*_1_^α^(0), *r*_2_^α^(0)}, these objects are then propagated according to the dyn-ICWF equations of motion in eqs 29 and 31.

To achieve converged results, we choose the size of the simulation box to be 150 × 150 au^2^ with a homogeneous grid consisting of 500 grid points in each direction. The fourth-order Runge–Kutta algorithm was used to propagate the dyn-ICWF equations of motion with a time-step d*t* = 0.01 au, and the Moore–Penrose pseudo-inversion method with a tolerance of 10^–8^ was used to approximate the numerical inversion of the overlap matrix in eq 31.

In Figure 12, we show the one-body electronic density ρ_e_(*r*_1_, *t*), for two different initial momenta and final times, viz., *p* = 0.3 and 1.5 au and *t* = 1.8 and 0.85 fs. For *p* = 0.3 au, a very small number of CWFs ((*N*_*c*_, *M*) = (16, 1)) is already able to capture the correct dynamics quantitatively. In approaching the target atom with the larger momentum *p* = 1.5 au, the conventional mean-field method fails to describe the ionization process due to the lack of electron–electron correlation effects. This is in contrast with dyn-ICWF results, which qualitatively captures the correlated dynamics for a small number of CWFs (*N*_*c*_, *M*) = (64, 1).

### C.2 Laser-Driven Proton-Coupled Electron Transfer

We discuss here the convergence behavior of the dyn-ICWF method in capturing the laser-driven proton-coupled electron transfer described in Section 6.2. We suppose the system to be initially seating in the full electron–nuclear ground state, i.e., Ψ(*r*, *R*, 0) = Ψ^0^(*r*, *R*). This state is prepared using the imaginary-time version of the sta-ICWF method with ground-state CWFs only (i.e., *M* = 1)

60

The sta-ICWF provides as output the initial expansion coefficients ***C***(0) and the ground-state electronic and nuclear CWFs, ϕ^α^(*r*, 0) and χ^α^(*R*, 0), respectively. We then apply an external strong electric field, defined in Section 6.2 of the main text, and the coefficients and the CWFs are propagated using the dyn-ICWF equations of motion in eqs 29 and 31.

To achieve converged results, a grid [−9, 9] au with 301 grid points is chosen for the nuclear degrees of freedom. For the electron coordinates, the grid covers the interval [−75, +75] au with 250 grid points. The fourth-order Runge–Kutta algorithm was used to propagate the dyn-ICWF equations of motion with a time-step d*t* = 0.1 au, and the Moore–Penrose pseudo-inversion method with a tolerance of 10^–8^ was used to approximate the numerical inversion of the overlap matrix in eq 31.

By introducing the Born–Huang expansion of the molecular wave function, Ψ(**r**, **R**, *t*) = ∑_*n*_Φ_**R**_^(*n*)^(**r**, *t*)χ^(*n*)^(**R**, *t*), we then monitor the dynamics through the BO electronic state populations, *P*_*m*_(*t*) = ∫d**R**|χ^(*m*)^(**R**, *t*)|^2^, and the overlap integral of projected nuclear densities evolving on different BOPESs, *D*_*nm*_(*t*) = ∫d**R**|χ^(*n*)^(**R**, *t*)|^2^|χ^(*m*)^(**R**, *t*)|^2^. These quantities can be written in terms of the dyn-ICWF basis by re-expressing the adiabatic nuclear components as

61

In Figure 13, we show dyn-ICWF results for (*N*_*c*_, *M*) = (16, 1). This very small number of CWFs, even if associated with large deviations across different stochastic particle placements, is able to capture nearly quantitatively both the adiabatic populations and the decoherence indicator. This result demonstrates that the dyn-ICWF technique achieves quantitative accuracy for situations in which the mean-field theory drastically fails to capture qualitative aspects of the dynamics using 3 orders of magnitude fewer trajectories than a mean-field simulation.

### C.3 Interference Effects Near a Molecular Conical Intersection

We discuss here some of the technical details of the interference effect calculation demonstrated in Section 6.3. As in ref (102), we took an electronic spatial grid [−12, 12] au with 81 grid points and a nuclear grid [−1.5, 1.5] au with 51 grid points alongside a time step of d*t* = 0.02 au. The initial wave function was constructed on this grid, and the exact dynamics were propagated directly using a fourth-order Runge–Kutta integrator.

The time-resolved picture presents this problem as a fully nonequilibrium problem, where the system starts already in a nonsteady state, and so, the imaginary-time sta-ICWF cannot be applied here to prepare the initial wave function. Instead, we stochastically sample the initial probability density |Ψ_0_(***r***, ***R***)|^2^ with *N*_*c*_ trajectories {***r***^α^(0), ***R***^α^(0)} that are used to construct CWFs ϕ_*r*_^α^(***r***, 0) and ϕ_*R*_^α^(***R***, 0), as defined in eq 23. In this process, we respected the symmetry of the underlying initial state by symmetrizing the initial particle placement (and thereby complementarily symmetric slice CWFs) around the *R*_*y*_, *r*_*y*_ axes, meaning for each particle *R*^α^ = (*R*_*x*_^α^, *R*_*y*_^α^), we set *R*^α+1^ = (−*R*_*x*_^α^, *R*_*y*_^α^).

These CWFs ϕ_*r*_^α^(***r***, 0) and ϕ_*R*_^α^(***R***, 0) are then used to construct the ansatz in eq 30, i.e.

62

with an initial **C** vector that is obtained using

63

where **G** is the vector containing the overlap between the initial wave function and the CWFs, i.e.

64

Given **C**(0), and ϕ_1_^α^(*r*_1_, 0) and ϕ_2_^α^(*r*_2_, 0) for an ensemble of sampling points {*r*_1_^α^(0), *r*_2_^α^(0)}, these objects are then propagated according to the dyn-ICWF equations of motion in eqs 29 and 31.

In the dyn-ICWF, the pseudo-inverse tolerance for  was set to 10^–8^ and the evaluation matrix elements of the electron–nuclear interaction potential term of eq 40

65

was accelerated using a singular value decomposition (SVD) to break up the four index potentials *W*_en_(*r*_*x*_, *r*_*y*_, *R*_*x*_, *R*_*y*_) into a sum over electronic and nuclear two index vectors

66

By tossing out σ_*l*_ < 10^–4^, we found that we were able to retain the accuracy of this potential to within a numerically tolerable limit with a speedup in computation time at a factor between 3.6 and 4.3 depending on hardware. A cubic interpolation to a grid twice as fine was used to smooth the images of the nuclear density

In Figure 14, we show the first and second excited BOPESs associated with the extended Shin–Metiu model introduced in Section 6.3. dyn-ICWF results for *N*_*c*_ = {1024, 1600, 2400} are shown in Figure 15. Due to the finesse of the interference pattern and its fragility with respect to the symmetry of the problem, the number of CWFs required to reproduce quantitatively the exact dynamics is relatively high compared to previous examples in Appendices C.1 and C.2. And yet, note that while the *N*_*c*_ = 1024 result do not reproduce the interference pattern accurately, they do qualitatively capture the nuclear dynamics by avoiding the forbidden region surrounding the conical intersection. This is in contrast to the mean-field result (Figure 7 of ref (102)), which fails to capture this qualitative feature of the nuclear dynamics.

## D Implementing the ICWF Method

We discuss here the general workflow associated with the different versions of the ICWF method, as well as some general remarks concerning their scalability with respect to the number of degrees of freedom.

Figure 16 illustrates all possible situations of interest. State preparation using imaginary-time sta-ICWF for equilibrium states is described in the top-left panel. A number *N*_*c*_ of particle positions ***x***^α^(0) are sampled from educated guesses of the single-particle reduced probability densities. These positions are used to construct the Hermitian Hamiltonians of eq 8 and a number *M* of eigenstates. The randomly initialized vector of coefficients ***C***^γ^(0) of the Ansatz in eq 9 is then propagated in imaginary time until convergence according to eqs 12–14. At this point, any (equilibrium) property of interest can be evaluated using eq 15 and 48 and 49.

The simulation may continue if the perturbation of an external agent is included. Thereafter, state propagation can be carried out using real-time sta-ICWF (bottom left) or dyn-ICWF (bottom right). As explained in the main text, if one chooses to propagate according to the sta-ICWF equation of motion (eq 21) together with eqs 13 and 14, a sufficient number of excited CWF states, γ > 0 in eq 9, for ***x***^α^(0) covering some anticipated range of motion for the dynamics must be considered. Alternatively, in cases where the time-dependent wave function is expected to stray far apart from the initial sta-ICWF basis, one may choose to address the dynamics using a time-dependent CWF basis as in eq 30 and the corresponding equations of motion in eq 29, together with eqs 31 and 32 and 13 and 14. At this point, any (nonequilibrium) property of interest can be again evaluated using eq 15 together with eqs 48 and 49.

Finally, note that in cases where one aims to study a certain dynamics that is triggered by some predefined out-of-equilibrium initial state, obtaining the initial coefficients ***C***(0) is done through direct matrix inversion (top right). Afterward, the dynamics must be simulated using the dyn-ICWF algorithm described above.

Importantly, at this level of approximation, the ICWF method is a wave function approach. That is to say that, while the simple sum-of-product form that we employed for our ansatz in Figure 16 can be made more efficient by introducing a tensor network representation for the expansion coefficients (such as matrix product states or hierarchical Tucker formats), an exponential scaling is not expected to be circumvented without introducing any further approximation.

## Footnotes

a This has been discussed at length in the literature. A general and very elegant discussion on the various ways the body-fixed frame can be chosen is given in refs (75) and (76).

b Both procedures lead to identical equations of motion due to the only time-dependent variational parameter being the expansion coefficients.
